# Data-Driven Improvement of Local Hybrid Functionals: Neural-Network-Based Local Mixing Functions and Power-Series Correlation Functionals

**DOI:** 10.1021/acs.jctc.4c01503

**Published:** 2025-01-13

**Authors:** Artur Wodyński, Kilian Glodny, Martin Kaupp

**Affiliations:** Technische Universitát Berlin, Institut für Chemie, Theoretische Chemie/Quantenchemie, Sekr. C7, Straße des 17. Juni 135, Berlin D-10623, Germany

## Abstract

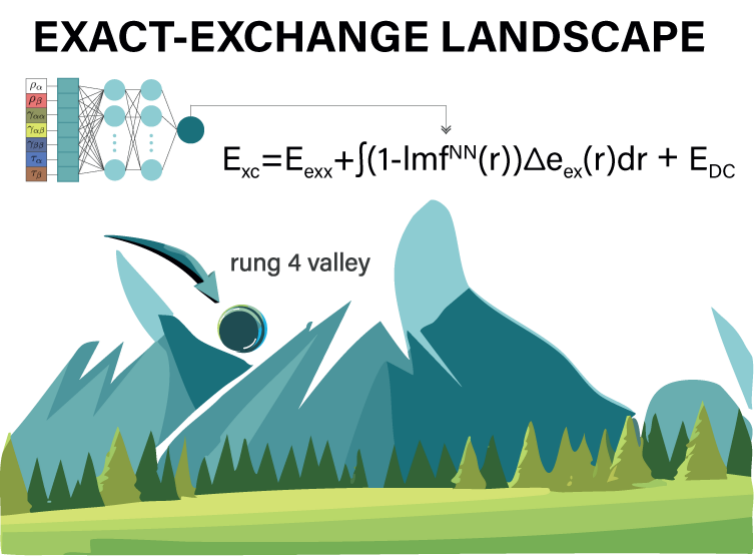

Local hybrid functionals (LHs) use a real-space position-dependent admixture of exact exchange (EXX), governed by a local mixing function (LMF). The systematic construction of LMFs has been hampered over the years by a lack of exact physical constraints on their valence behavior. Here, we exploit a data-driven approach and train a new type of “n-LMF” as a relatively shallow neural network. The input features are of meta-GGA character, while the W4-17 atomization-energy and BH76 reaction-barrier test sets have been used for training. Simply replacing the widely used “t-LMF” of the LH20t functional by the n-LMF provides the LH24n-B95 functional. Augmented by DFT-D4 dispersion corrections, LH24n-B95-D4 remarkably improves the WTMAD-2 value for the large GMTKN55 test suite of general main-group thermochemistry, kinetics, and noncovalent interactions (NCIs) from 4.55 to 3.49 kcal/mol. As we found the limited flexibility of the B95c correlation functional to disfavor much further improvement on NCIs, we proceeded to replace it by an optimized B97c-type power-series expansion. This gives the LH24n functional. LH24n-D4 gives a WTMAD-2 value of 3.10 kcal/mol, the so far lowest value of a rung 4 functional in self-consistent calculations. The new functionals perform moderately well for organometallic transition-metal energetics while leaving room for further data-driven improvements in that area. Compared to complete neural-network functionals like DM21, the present more tailored approach to train just the LMF in a flexible but well-defined human-designed LH functional retains the possibility of graphical LMF analyses to gain deeper understanding. We find that both the present n-LMF and the recent x-LMF suppress the so-called gauge problem of local hybrids without adding a calibration function as required for other LMFs. LMF plots show that this can be traced back to large LMF values in the small-density region between the interacting atoms in NCIs for n- and x-LMFs and low values for the t-LMF. We also find that the trained n-LMF has relatively large values in covalent bonds without deteriorating binding energies. The current approach enables fast and efficient routine self-consistent calculations using n-LMFs in Turbomole.

## 1 Introduction

Kohn–Sham density functional theory (KS-DFT) remains the dominant workhorse of electronic-structure theory in quantum-chemistry, solid-state physics, and material sciences.^1−4^ Increasing the accuracy of approximate exchange-correlation (XC) functionals while maintaining low computational cost constitutes the holy grail in the development of KS-DFT. Following Perdew’s ladder^5^ hierarchy of density functional approximations (DFAs) from the local (spin) density approximation (L(S)DA, rung 1) via the generalized gradient approximation (GGA, rung 2) to meta-GGA functionals (rung 3), the hyper-GGA rung 4, and the fully nonlocal functionals on rung 5, both complexity and computational effort tend to increase. This is often accompanied by a reasonable chance that the achievable accuracy can be enhanced as well. Rung 4, where exact exchange (EXX) enters the picture, appears to provide the largest room for improvement at still affordable computational cost, certainly so in a molecular setting. Initiated by the introduction of global hybrid functionals^6,7^ (GHs) with a constant EXX admixture, various notable branches of hybrid functionals have evolved: range-separated hybrids^8,9^ (RSHs) vary the admixture in interelectronic distance space, local hybrids^10,11^ (LHs) in coordinate space. Various combinations of these ideas exist, such as range-separated local hybrids^12,13^ (RSLHs), locally range-separated hybrids^14,15^ (LRSHs), and even most recently locally range-separated local hybrids^16,17^ (LRSLHs), which all bring in their own advantages and complexities. We have recently shown that LHs and RSLHs can be augmented by correction terms for strong correlations^18−21^ which opens unprecedented pathways out of the usual zero-sum game^22,23^ between delocalization and strong-correlation errors. We are firmly convinced that these types of constructions, which depend on the EXX energy density, will be an important ingredient of the future of KS-DFT.
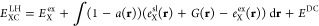
1

The focus of this work is on LHs, which locally mix EXX and semilocal exchange-energy densities in coordinate space to model nondynamical correlation (NDC) in a flexible way, governed by a so-called local mixing function *a*(**r**) (LMF). We expect, however, that the developments reported in this work can also be extended to RSLHs, and potentially even to rung 5 functionals. A wide variety of LMFs has been proposed, and we refer the reader to our 2019 review^11^ for an overview, and to refs. (23 and 24) for more recently proposed models. We note in passing that strong-correlation corrections also modify the LMF in important ways,^19^ but we will explicitly not examine such terms in this work. A particularly useful expression of an LH functional is shown in eq 1, where we can identify full exact exchange on the left, a crucial middle NDC term, and a largely dynamical correlation (DC) contribution on the right. We refer to a recent detailed discussion regarding the interpretation of the middle term as an NDC contribution.^24^ Its integrand multiplies the complement of the LMF, 1 – *a*(**r**), by the difference between semilocal exchange and EXX energy densities,  and , respectively. It can contain a so-called calibration function (CF), *G*(**r**), to mitigate the so-called gauge problem of LHs that is due to the ambiguity of exchange-energy densities.^11^ However, we have recently shown that the gauge problem can also be suppressed by a suitable form of LMF.^24^ Indeed, we will show that this extends also to the type of LMFs proposed in this work, obviating the need of a CF.

The starting point of this work will be the LH20t^25^ functional. LH20t has also been the basis for the more recent ωLH22t^13^ RSLH, and for subsequent strong-correlation-corrected LHs^19,20^ and RSLHs.^21^ While LH20t has been the first LH that outperformed the existing GHs for the large GMTKN55 main-group energetics suite, and it provides very promising accuracy in many other areas for ground and excited states, we have ample reason to believe that the simple so-called “t-LMF” used also in LH20t is not the optimum form one may achieve. On one hand, we can point to the still better performance of the ωB97M-V^26^ RSH or the very recent highly parametrized B22plus^27^ or CF22D^28^ GHs for the same test set (several of our recent RSLHs also perform better). On the other hand, we know that the scaled t-LMF violates a number of exact constraints, such as the high-density coordinate scaling limit,^29^ the one-orbital limit^11^ or the asymptotic limit.^11,29^

But even with an LMF that fulfills these exact constraints, we are not guaranteed to achieve higher accuracy for many quantities, due to the fact that an insufficient number of exact constraints is currently available to fix the form of a rung 4 functional in general and the LMF of an LH in particular: the middle term in eq 1 is supposed to model left–right correlation in chemical bonds while keeping delocalization errors low. But there are no exact constraints that would tell us how the LMF should look like in the bonding and generally in the valence region to achieve these goals. This is a major aspect this work addresses. The absence of first-principles guidelines to construct these parts of an LMF suggests that a data-driven approach may provide improved LMFs for valence properties, such as chemical energy differences. We therefore introduce so-called n-LMFs constructed as relatively shallow neural networks^30,31^ (NNs).

Machine learning (ML) within the context of DFT has exploded over the past decade, and the literature is already too extended to review it here in its entirety. We can roughly distinguish (a) approaches that use ML based on KS-DFT data to parametrize cheaper approaches, such as force fields^32^ or orbital-free DFT,^33^ (b) Δ-ML to improve upon DFT accuracy with respect to high-level ab initio data,^33−38^ and (c) learning the entire XC functional or aspects of it.^28,33,39−48^ This work belongs to the latter category, as we will use an NN to train only one aspect of the XC functional, namely the LMF of a LH. We draw some inspiration from but contrast our work to the recent DM21 functional.^40^ DM21 is a complete deep-NN rung 4 functional. It achieves remarkably low delocalization and strong-correlation errors and also is relatively competitive for test sets like the GMTKN55 suite we will examine here. But it is a completely black-box functional that allows almost no analysis or understanding (it also appears to suffer from some numerical instabilities affecting SCF convergence).^49^ Notably, the input features of DM21 qualify it as a RSLH. Modeling only the LMF of an otherwise human-designed LH in the present work should provide much more insight: the LMF is a relatively simple real-space function limited to the interval between 0 and 1 (between −1 and 1 in strong-correlation LHs,^19^ which will not be covered here). We can examine it in a straightforward manner graphically for different molecules to enhance our understanding.

Our initial approach will be to just replace the t-LMF in LH20t by an n-LMF while keeping the other aspects of the functional unchanged. This should enable us to see the effect of the LMF in a particularly direct way. The n-LMF will be trained against atomization energies and reaction barriers. The second improvement we will report here is to replace the B95c DC part of LH20t (see below) by a more flexible B97c-type^50^ power series expansion. The reason is that we found the B95c^51^ parametrization of the recent LH24x functional^24^ (based on an x-LMF) to differ substantially from that of LH20t, and this mattered for the performance, in particular in the context of noncovalent interactions (NCIs) in the interplay with dispersion corrections. Combining the new n-LMF for the NDC term with the more flexible optimized B97c-style DC contribution results in the LH24n functional that will be a central result of this work and provides substantial enhancements over LH20t for GMTKN55^52^ performance, without needing a CF! It should be noted that the data used for optimization and training cover neither the core nor the asymptotic regions of a molecule. We should therefore expect no improvements in properties depending on these regions, and at this point we will focus entirely on typical chemical energy differences. Moreover, we have so far not used strong-correlation data in the parametrization, and we do not yet consider RSLH forms.

## 2 Theory

As we use LH20t^25^ as our starting and reference point, eq 2 shows its explicit form:
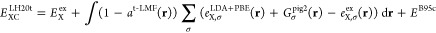
2(in contrast to eq 1 above, here we show the explicit summation over the spin channels  in the integrand of the middle term).

The t-LMF,^10,25,53^ is
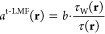
3where  is the von Weizsäcker kinetic-energy density and  the Kohn–Sham kinetic-energy density, all taken over both spin channels (“common t-LMF”^11^), and *b* is a scaling factor.

Full EXX is defined as
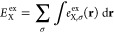
4where  is the (spin-resolved) EXX energy density
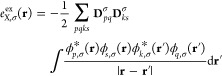
5defined using one-particle density matrices (**D**^σ^) within the atomic orbital basis (ϕ).

 is the DFT GGA^54^ exchange energy density (with downscaled GGA enhancement). The semilocal CF, , is a so-called partial-integration-gauge construction to second order (pig2).^55^ See refs. (11, 55) for more details.

As DC part, LH20t uses the B95c^51^ meta-GGA correlation functional, given by
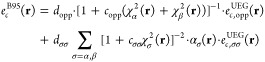
6with four optimized parameters (*d*_opp_, *d*_σσ_, *c*_opp_, *c*_σσ_).  is the reduced density gradient and  is used as a self-correlation correction for the same-spin contribution.

### 2.1 Construction and Training of a Neural-Network LMF

Training and use of an NN model for the LMF (“n-LMF”) are done in two separate phases. The training of the LMF is done in a post-SCF manner in a locally developed Python code connected to the TensorFlow library.^56^ The input features of the LMF are transferred from the Turbomole code^57−59^ and are based on a human-designed functional, ensuring that we have well-defined descriptors. The weights of the trained n-LMF are subsequently transferred back to Turbomole, where standard self-consistent DFT calculations with the n-LMF can then be conducted.

In the development of our n-LMF model, a multilayered architecture was constructed to perform a series of matrix operations and nonlinear activations.^60^ The input layer of the network receives a vector, , representing 7 distinct features. The input features include the spin-resolved electron density (2 features), the squared norm of the gradient of the spin-resolved electron density (3 features, , , ), and the spin-resolved kinetic energy density (2 features).

The features are transformed prior to being input to the neural network to ensure they are appropriately normalized for effective learning (see, e.g., discussion in ref. (61)). This “squashing” transformation, applied individually to each feature **x** is defined as
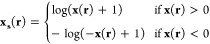
7The first hidden layer employs the weight matrix  and bias vector . The output of this layer is
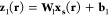
8
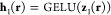
9with GELU (Gaussian Error Linear Unit)^62^ applied element-wise. The differentiability of the GELU activation function, expressed by
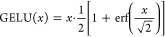
10is crucial not only for weight optimization during training of the weights but also for ensuring stability in the SCF process for the trained functional.

The second hidden layer utilizes the weights  and biases , processing the output from the first hidden layer to produce:
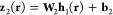
11
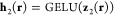
12The network’s output is generated by the output layer through the weight matrix  and bias **b**_3_. The final neuron uses a sigmoid activation function σ, resulting in
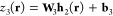
13
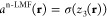
14The sigmoid function,^60^ defined by the equation  smoothly maps input values to the range (0, 1), making it ideal for construction of the LMF.

Derivatives of *a*^n-LMF^ with respect to **x**(**r**) are straightforwardly derived using the chain rule:
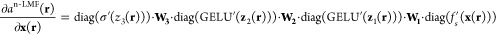
15where  is the derivative of the element-wise transformation function used for the input features.

To preserve spin symmetry in the NN, which receives spin-resolved features, the features are passed through the network twice during both training and prediction. In the first pass, the features are presented in the normal order (α, β), and in the second pass, the order is reversed (β, α). The final prediction of the NN is then obtained by averaging the results from both passes. This approach ensures that the model treats spin symmetrically and avoids bias toward a specific spin configuration. This method has been proposed originally in ref. (40) and has been shown to be effective in maintaining spin symmetry in NN models.

#### 2.1.1 NN Architecture Selection

The NN architecture we selected is deliberately small and not excessively deep, consisting of only 64 neurons per layer over two hidden layers. This choice was influenced by the relatively limited amount of labeled data used, with 276 energy differences in the training set, which consisted of the W4-17 atomization energy and BH76 reaction barrier test sets in equal weights. During the model selection process, we experimented with larger architectures, including a model with 128 neurons in each of *three* hidden layers. However, these larger models exhibited a tendency to overfit the training data. That is, larger models were more effective at minimizing the loss function for W4-17 and BH76, but this did not translate into superior performance when evaluated on the much larger GMTKN55 benchmark. In addition, while early stopping after around 2000 epochs did improve significantly validation performance of these larger models, the results still did not surpass those obtained with the chosen smaller architecture. Consequently, the simpler, shallower model (employing 4737 trainable parameters) was deemed more appropriate for our specific data set (see Tables S1 and S2 for different choices of the number of neurons and layers for the n-LMF based on a hyper-meta-GGA set of features; cf. also below).

During the early stages of this work we also conducted tests with various activation functions for the hidden layers, including ReLU,^63^ GELU,^62^ tanh (see, e.g., ref. (64)), and sigmoid^60^ functions. ReLU and GELU produced relatively similar results in terms of performance. However, ReLU exhibited some convergence issues within the subsequent SCF process for certain molecules, which limited its reliability in this context. The tanh activation function also yielded comparable results to ReLU and GELU, while the sigmoid function performed overall slightly worse (see Tables S1 and S2 for comparisons of GELU and tanh for an n-LMF based on a hyper-meta-GGA set of features).

We also explored the use of various features in the construction of the LMF. At an early stage, the density Laplacian was abandoned as a feature due to convergence issues observed during subsequent SCF calculations. We conducted detailed evaluations using the EXX energy density as one of the features, which provides a hyper-meta-GGA-type LMF.^65^ This did not alter the quality of the LMF notably. However, it may generate some complications for further implementations, e.g., in a TDDFT framework, as the functional becomes nonlinearly dependent on the EXX energy density. We therefore decided to currently focus on a typical meta-GGA set of features for the n-LMF. While further, more systematic tests are conceivable and may provide further insights, we are confident to have identified the best selection of activation functions for a stable and effective process.

### 2.2 Construction of the Initial LH24n-B95 Functional

**Figure 1:**
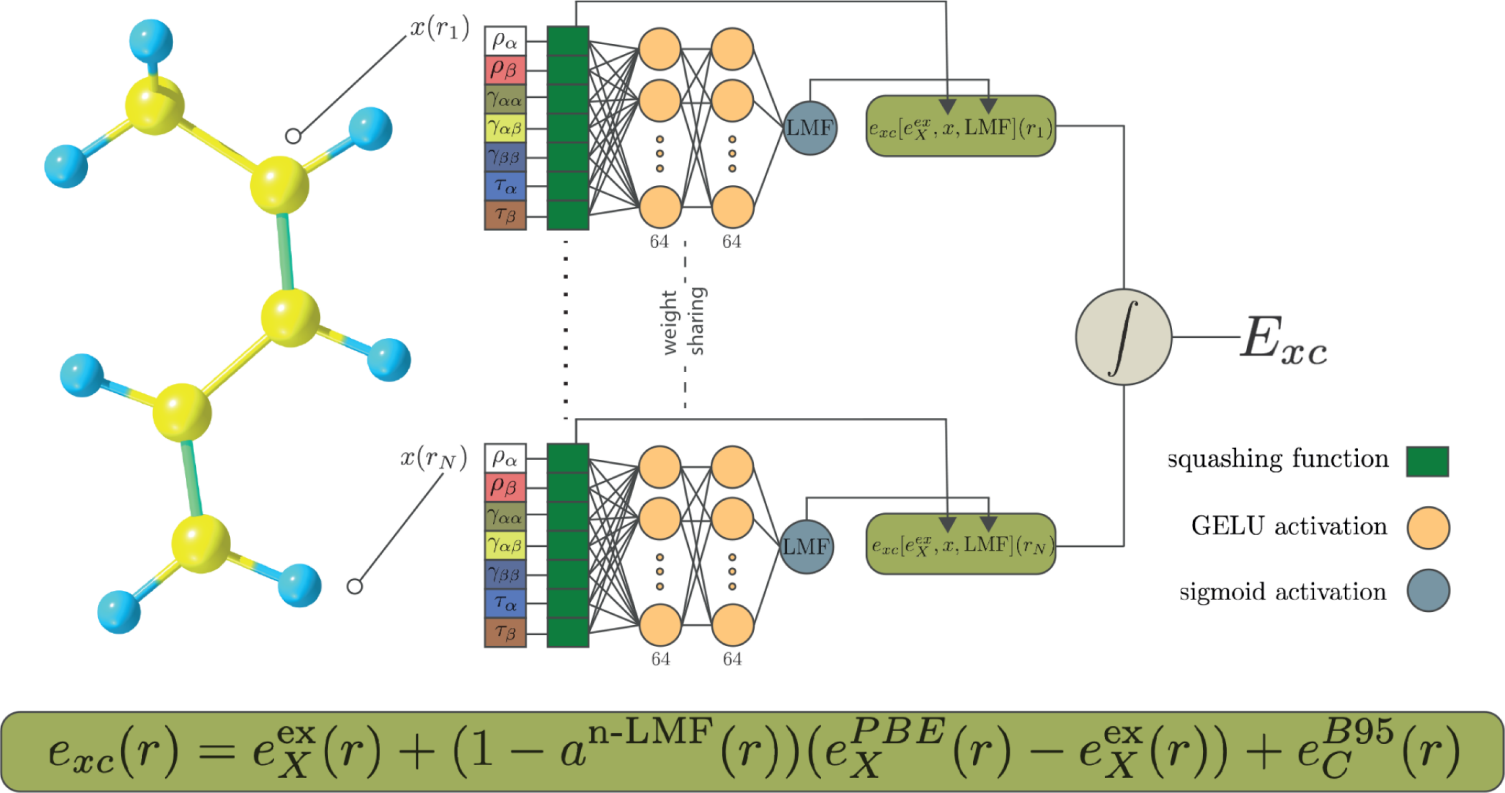
Illustration of the procedure leading to the LH24n-B95 functional.

The first step in the present development of new LHs starts from LH20t and replaces the t-LMF *a*^t-LMF^(**r**) by the n-LMF *a*^n-LMF^(**r**) described above, trained in the presence of the other unchanged parts of the LH (cf. eq 2 above). The CF  is omitted in this process, as it turned out early on that the n-LMF successfully suppresses the gauge problem (as found previously for an x-LMF),^24^ and therefore no CF is required. We will analyze the reasons for this observation in detail in the Results section. Another small modification compared to LH20t is that we fix the coefficient of PBE exchange gradient corrections to full 1.0 rather than optimizing it to a smaller value as done in LH20t (see above). Then the LH24n-B95 functional can be described as
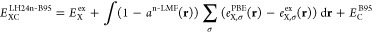
16The process that leads to this functional is illustrated in Figure 1.

The parametrization of B95c (see eq 6) is important in this context. We noted previously that the  optimized for LH20t is larger than the original parameter () found by Becke.^51^ This reduces somewhat the same-spin DC contribution, which improved performance for, e.g., noncovalent interactions (NCIs) when combined with dispersion corrections. When using Becke’s original value with the LH, one can observe even a deterioration of intramolecular NCIs within the GMTKN55 test suite upon adding dispersion terms (see Tables S1 and S1 for results with a hyper-meta-GGA-based n-LMF). We therefore decided to retain the entire B95c parametrization found for LH20t also for LH24n-B95. Attempts to simultaneously optimize B95c and LMF parameters led to nonphysical results. This may have to do with the high flexibility of the n-LMF, which tends to absorb any influence of the DC contribution. This is supported also by a consistent performance improvement of the n-LMF compared to the t-LMF in our initial evaluations when used with different B95c parameters (see Tables S1 and S2 for results with a hyper-meta-GGA-based n-LMF). Retaining the B95c parameters from LH20t also simplifies comparisons between the two functionals, putting the focus squarely onto the LMF. LH24n-B95 has been further augmented by optimizing DFT-D4 dispersion corrections in the prescribed way against the standard S22x5, S66x8, and NCIBLIND test sets, resulting in LH24n-B95-D4. All parameters of the functionals of this work, apart from the n-LMF itself, are summarized in Table S3. The algorithm for reading the file with the weights and biases for the n-LMF is also provided in Supporting Information. The actual weights and biases are available in a separate file under 10.5281/zenodo.13969761.

### 2.3 Power Series Expansion of B97c Correlation, toward the LH24n Functional

B97-type power-series expansions for both exchange and correlation have become important for many literature functionals^26,66−68^ starting with Becke’s original work.^50^ The essential initial idea has been to attempt to generate as flexible GGA functionals as possible. Later adaptations extended the B97c correlation functional to a meta-GGA framework by adding self-correlation corrections. Our focus here is just on B97c-type correlation with such self-correlation corrections only for the same-spin part (employing α_σ_(**r**) as in B95c, cf. eq 6) as a more flexible alternative to B95c used so far. We thus consider a correlation-energy density of the form
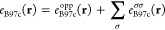
17
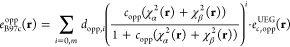
18
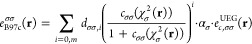
19B95c can be seen as a special case of such a model. If  is expanded only up to *m* = 1,
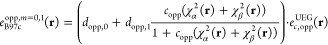
20and we set *d*_opp,1_ = −*d*_opp,0_ = *d*_opp_, we reproduce the B95c opposite-spin contribution:
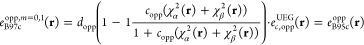
21

A more nuanced relationship between B95c and B97c emerges for the same-spin contributions. Expansion of *e*_B97c_ up to *m* = 2 leads to
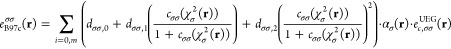
22If  and , then
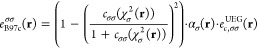
23

After rewriting we find
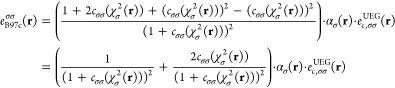
24That is, we get a mixed-power contribution, which would have to be removed to arrive at B95c proper.

We have replaced B95c by a reoptimized B97c model starting from LH24n-B95 and thereby obtained the final LH24n functional. The linear B97c parameters have been optimized by minimizing the WTMAD-2 value of the full GMTKN55 test suite in nonself-consistent calculations with LH24n-B95 orbitals and in the presence of the unaltered D4 contributions from LH24n-B95-D4 (see above), keeping the n-LMF unchanged except for a simple scaling (see Results section). We thereby arrive at LH24n-D4. For comparison, we also replaced B95c by B97c in LH20t, keeping the CF and D4 terms unchanged and optimizing only the linear parameters of B97c and the scaling factor *b* of the t-LMF (eq 3). We will denote this as LH24t (and LH24t-D4).

## 3 Computational Details

The NN optimization of the n-LMF was performed with the TensorFlow^56^ library in a local python code. The NN training used input features (see Theory section) obtained with a local version of Turbomole 7.8^57−59^ based on the scLH23t-mBR-P^20^ LH, def2-QZVPPD^69^ basis sets, and Turbomole’s “universal” auxiliary basis sets^70^ for the RI-J approach^71,72^ to the Coulomb integrals, with gridsize 3. The features consisted of the density, the norm of the density gradient, and the kinetic-energy density. Each grid point was processed using a multilayer perceptron^30^ (MLP) with shared weights (see discussion in Section 2.1 and Figure 1). The output layer was employed in eq 16 to predict the final XC energy density for each atom or molecule in the BH76^73,74^ and W4-17^75^ data sets. Integration of the predicted XC energy densities was done using Becke’s partitioning scheme.^76^ The integrated energies were labeled with the reference values obtained from these databases. The mean absolute error (MAE) was calculated for each data set and integrated into the loss function with equal weights for the two sets.

This approach of labeling with respect to the integrated reaction energies is analogous to the procedure used for training the DM21 functional.^40^ One might consider going beyond this “Global Energy Loss” (GES) and using energy densities instead (“Local Energy Loss”, LES).^77^ While we consider this for future work, it requires high-level energy densities for training. Our training regimen encompassed 5000 (8000 in selected examinations) epochs with weights updated at the end of each epoch. Taking into account a limited reproducibility and the influence of the chosen starting point, several training runs with different seeds in the GlorotUniform^78^ weights initializer were performed, and the overall best result was chosen. The sensitivity of the optimization to the choice of initial conditions and limited reproducibility is demonstrated in Figure S1, which presents SCF results (see below) for various network models using a hyper-meta-GGA-type n-LMF. While we finally settled on a meta-GGA-type n-LMF, we expect a similar variance of 0.5 kcal/mol or less for most cases.

The trained n-LMF was then integrated into a local version of the Turbomole^57^ software together with analytical derivatives defined by eq 15. This allowed for practical and routine self-consistent calculations within Turbomole in a standard workflow. For all t-LMF- or n-LMF-based functionals of this work, self-consistent calculations were done for the entire GMTKN55^52^ test suite. This involved either the B95c^51^ correlation functional or the described B97c-style power series.^50^ For the nonself-consistent optimization of the linear parameters of the latter (see above), a BFGS algorithm^79−82^ has been used, minimizing the WTMAD-2 value of the full GMTKN55 database with LH24n-B95 orbitals, in the presence of the D4 dispersion corrections optimized previously for the B95c-based variant of a given functional.

Calculations for the W4–17 and BH76 sets were done with def2-QZVPPD^69^ basis sets and gridsize m3. Computations on GMTKN55 used def2-QZVP^83^ basis sets (with augmentation by diffuse functions for a few subsets, as described in ref. (52)) and gridsize m4, consistent with literature procedures. Additional evaluations of LH24n-B95-D4 and LH24n-D4 on real-world organometallic transition-metal reaction energies (MOR41^84^ and ROST61^85^) and barrier heights (using a modified MOBH35^86−89^ subset called MOBH28),^90^ def2-QZVPP^83^ basis sets, along with the def2-ecp-type Stuttgart-Dresden scalar-relativistic pseudopotentials for 4d and 5d transition-metal atoms,^91^ gridsize m5, and the RI approximation were employed.

The two-electron integrals required for EXX energy densities were computed by seminumerical integration,^92−95^ with standard screening settings provided by Turbomole. In most cases Turbomole’s “universal” auxiliary basis sets^70^ were employed for the RI-J approach^71,72^ to the Coulomb integrals. Convergence criteria for SCF convergence were set to 10^–7^ Hartree. We generally observed no appreciable SCF convergence issues, even for challenging systems with stretched bonds contained within GMTKN55, indicating that the SCF process with the transferred n-LMF was stable under the various conditions imposed by the different data sets and computational settings.

## 4 Results

### 4.1 Training of the n-LMF and Resulting Atomization Energies and Barrier Heights

As done usually in NN-training for DFT functionals, we use a post-SCF approach for the n-LMF (see above). SCF-based training is done rarely so far^96−98^ in spite of its possible advantages for training dynamics and regularization, due to computational challenges, in particular for larger data sets (we note recent efforts for the DM21^40^ functional regarding SCFloss to mitigate such issues). As transferability of a post-SCF training is of course a possible issue, we have performed SCF calculations with the post-SCF n-LMF parameters every 400 epochs to monitor the effects of self-consistency. Ultimately, the n-LMF and functional exhibiting the best performance in SCF calculations was selected for final analysis, to ensure good applicability in practice.

**Figure 2:**
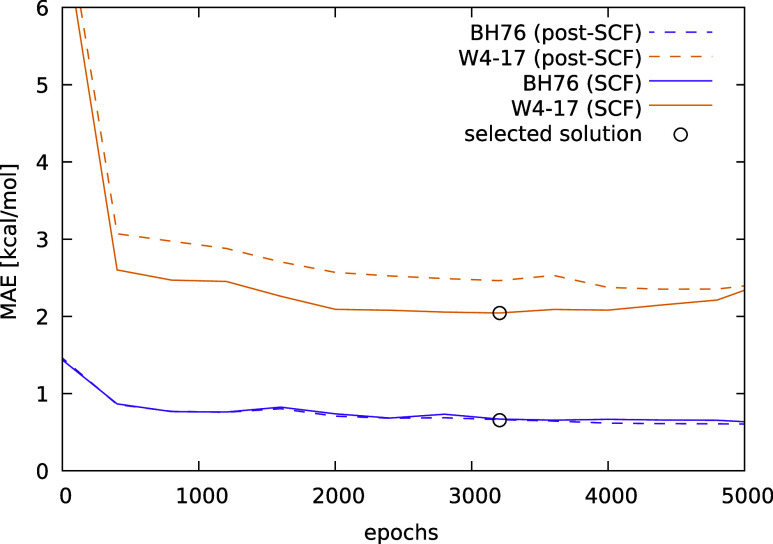
Optimization and comparison of post-SCF and SCF results for W4-17 and BH76 during n-LMF training (for LH24n-B95). The evolution of the loss values across the training epochs is shown.

Figure 2 illustrates the optimization trajectory and the comparative analysis between post-SCF and SCF results. Within the initial few hundred epochs, the loss values decreased below 3 kcal/mol for W4-17 and below 1 kcal/mol for BH76. Continued relaxation of the neural network led to gradual improvements, with post-SCF calculations attaining loss values below 2.5 kcal/mol for W4-17 and approximately 0.7 kcal/mol for BH76. Differences between post-SCF and SCF MAEs for BH76 were found to be negligible. On the other hand, the SCF MAEs were appreciably lower than the post-SCF ones for the W4-17 atomization energies. Notably, after a plateau observed between 2000 and 3500 epochs, a slight increase in SCF loss values emerged, potentially attributable to overfitting or challenges in transferring post-SCF optimizations to SCF computations. In certain optimization scenarios, a more pronounced increase in loss values at higher epoch counts was observed, indicating that incorporating SCF training methodologies (or utilizing SCFloss) might be advantageous. For the time being we applied an approach inspired by early stopping techniques based on the SCF MAE. That is, we selected the lowest MAE of the SCF solutions and avoided further iterations that increase complexity without improving accuracy in a routine workflow.

Table 1 summarizes the selected SCF results for the W4–17 and BH76 data sets with the final LH24n-B95 functional in comparison with LH20t and some further LHs and RSLHs from our group (these were also optimized for comparable barrier-height and atomization-energy test sets). LH24n-B95 improves the W4–17 MAE by about 30% compared to these other functionals. For BH76, the MAE is even lowered by ca. 60% over the LHs and still by about 30% over the best-performing RSLHs, which benefit from their long-range EXX admixture. Note that ωLH23td corrects ωLH22t by a delocalization-error correction term in the LMF, while ωLH23tdE additionally adds strong-correlation corrections. None of this applies to LH24n-B95, and thus the improvements for both barriers and atomization energies are entirely due to the use of an n-LMF. We may anticipate that use of an n-LMF for RSLHs may provide even further improvements. We currently investigate this possibility in our group. The most apt comparison at this point is of course between LH20t and LH24n-B95, clearly showing the striking effectiveness of replacing the t-LMF by the n-LMF for both training subsets. We find the dramatic improvement for BH76 particularly notable and will analyze this further below.

**Table 1:** Self-Consistent Performance of LH24n-B95 for the Optimization Test Sets (MAEs in kcal/mol) in Comparison with Some LHs and RSLHs Based on a t-LMF

	BH76	W4-17
LH20t	1.66	3.12
scLH22t	1.57	3.51
ωLH22t	1.08	2.93
ωLH23td	0.97	2.94
ωLH23tdE	0.95	3.09
LH24n-B95	0.67	2.04

### 4.2 Analysis of the n-LMF in Real Space

**Figure 3:**
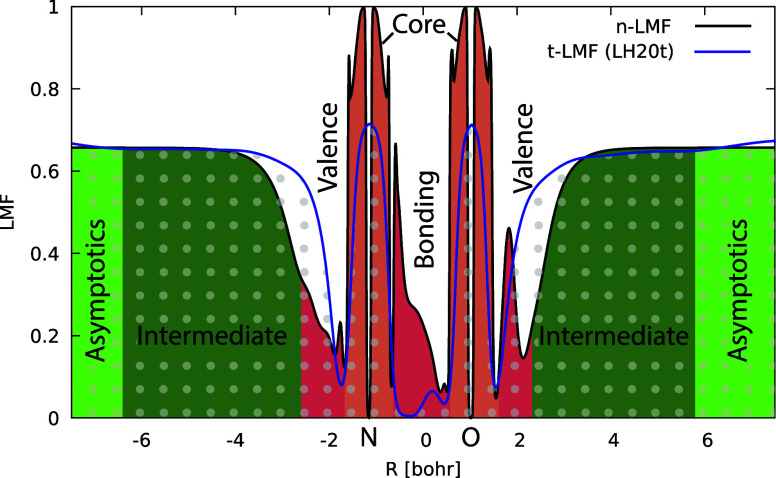
Comparison of the shape of the LMF for the NO molecule along the bond axis. The black line and colored areas represent the n-LMF trained in this study, while the blue line and dotted areas correspond to the t-LMF. The figure highlights key regions: the core region, the valence region within and outside the bond, and the intermediate region leading to the asymptotic behavior. See also ref. (11) and work cited therein.

As discussed in the introduction, limiting the NN training to just the LMF of a human-designed flexible but well-defined LH, rather than deep-learning the entire functional as for example in the case of DM21, opens the possibility to examine the shape of the resulting LMF. This can be done in comparison with other known LMF models. Figure 3 illustrates the shape of the n-LMF for the NO molecule along the bond axis in comparison with the t-LMF of LH20t (plots for more diatomics are shown in Figures S2–S4). In Figure 3 we use a color shading of typical spatial regions, such as the core region, valence areas within and outside the bonding region, an intermediate region, and the asymptotic region far from the nuclei in a real-space sense. We have to reiterate that the current training sets for the n-LMF do not cover any data determined by the asymptotics or the core part. It is nevertheless interesting to see the similarity of the asymptotic plateau of the n-LMF and the scaled t-LMF. For the t-LMF this value is of course determined by the scaling parameter *b* = 0.715 in LH20t (eq 2), which arises from the optimization for atomization energies and barriers, just as in the training of the more flexible n-LMF. Of course the correct asymptotic behavior should lead to full EXX admixture far from the nuclei, but this aspect is so far not reflected at all by the training data. Similarly, we will refrain from discussing the core region in any detail, except for noting that the training did result in large values in the vicinity of the nuclei but a sharp drop directly at the nuclei.

Given the training for valence energy differences, the valence region and the intermediate region toward the asymptotics are more interesting to analyze. Regarding the latter aspect, the n-LMF reaches the plateau later than the t-LMF albeit with a similar slope. Notably, the n-LMF exhibits additional narrower peaks on each side outside the bonding region before this rise, much more pronouncedly on the side of the more electronegative oxygen atom. This seems to hold true also for other heteronuclear diatomics like CO (see Figure S2). We note in passing that the so-called density-overlap regions indicator (DORI),^99,100^ which also has been used to construct an LMF,^101^ exhibits similar valence features in even sharper form. The bonding region appears most important given the nature of the training data. The t-LMF is known to go to zero at the bond-critical point (BCP)^102^ due to the vanishing density gradient included in τ_*W*_, while local maxima left and right of the BCP tend to develop upon bond stretching. The increase of these maxima for the t-LMF upon bond stretching, providing some compensation for exaggerated left–right correlation by semilocal functionals, has been connected to the good performance of t-LMF-based LHs for barriers (e.g., in comparison with s-LMFs).^103^ Given that the present n-LMF performs even much better for barriers, it is notable that in covalent bonds it actually produces more EXX admixture near the BCP. In heteronuclear bonds, the LMF seems to be larger near the less electronegative atom (Figure S2). Interestingly, for the n-LMF the maximum *around* the BCP increases and becomes wider upon bond stretching, while the additional valence peaks closer to the nuclei are less affected (see Figures S3 and S4). This apparently provides a different mechanism to improve reaction barriers. It seems important that relatively large EXX admixtures in the bond region provided by the n-LMF do not appear to affect atomization energies in an adverse way, as these are also improved (see above). Overall the shape of the n-LMF in the bonding region differs remarkably from that of the t-LMF. It seems clear that *particularly low* EXX admixtures in the bond are not required to provide good atomization energies, in spite of the role of semilocal exchange in an LH to model left–right correlation.

The t-LMF degenerates to a constant in one-orbital regions, e.g., for the entire H_2_ ground state or “behind” hydrogen atoms in X-H bonds (cf. Figure S2). This has been discussed as a potential shortcoming of the t-LMF in different contexts.^11^ The present n-LMF shows interesting structure for such regions (Figure S2), which may contribute to the improved behavior for many energy differences, in particular for atomization energies or barriers involving hydrogen atoms. We do indeed find a more pronounced improvement with LH24n-B95 compared to LH20t for such cases among the atomization energies of the W4-11 set (from 1.64 to 1.35 kcal/mol involving bonds to hydrogen, only from 4.76 to 4.67 kcal/mol for the non-hydrogen cases).

### 4.3 Suppression of the Gauge Problem by the n-LMF

For other LHs and RSLHs, in particular those based on the t-LMF (which are the most closely examined ones so far), unphysical NDC contributions arise from a mismatch of the semilocal exchange and EXX energy densities. They manifest most clearly as dramatically too large Pauli repulsions in potential-energy curves for noncovalent interactions (NCIs), e.g., in case of noble-gas dimers like Ar_2_ (see for example discussion in ref. (11)). These gauge artifacts can be suppressed by a suitably optimized CF. We have recently found that gauge problems in such curves appear to be virtually absent even without a CF when employing a so-called x-LMF^24^ based on a ratio between the same semilocal and EXX energy densities. Detailed analyses of the reasons for these observations were provided by analyzing the middle term of eq 1. We find the trained n-LMF to feature similar advantages, even though the training data do not include notable NCIs.

**Figure 4:**
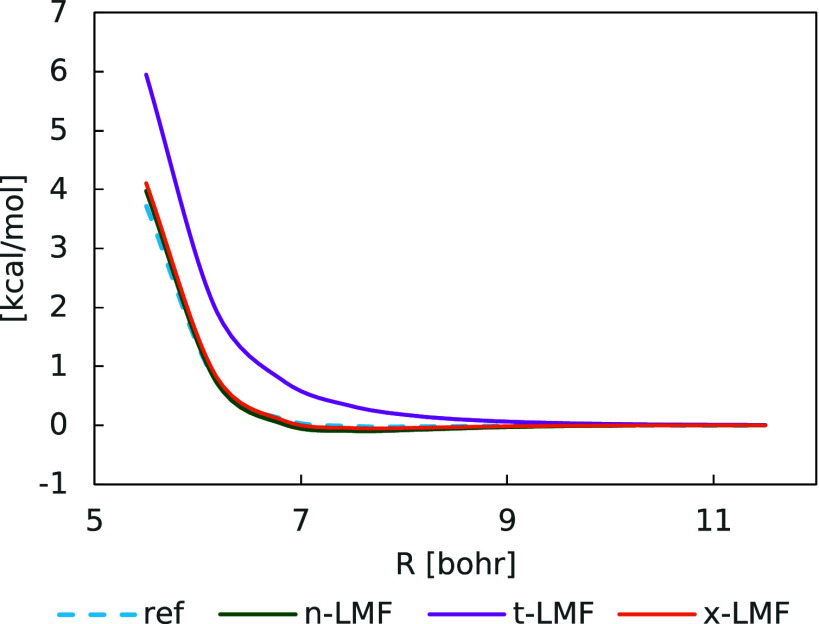
Comparison of dissociation curves for the argon dimer for different LMFs without CF. For simplicity of comparison, the curves for all LHs and the HF + B95c reference curve were obtained with an identical B95c parametrization (see text).

We typically illustrate the gauge problem and its suppression (either by a CF or by the choice of LMF) by comparing curves like that for Ar_2_ in Figure 4 with reference curves using full EXX and the given DC functional (for simplicity of comparison, the x-LMF curve is obtained here with the same B95c parametrization as LH24n-B95 and LH20t without CF). This choice is motivated by the assumption that NDC should be absent in such a system. The plot shows the too repulsive curve obtained with LH20t when omitting the CF (the reference curve is HF + B95c). As found in ref. (24), the excessive Pauli repulsions arise from unphysically positive local contributions in the middle term of eq 1 because the semilocal exchange-energy density is less negative locally than the EXX one. Figure 4 shows that LH24x based on an x-LMF does not exhibit such artifacts but rather provides an only very slightly too attractive curve. Interestingly, the curve obtained with the n-LMF (i.e., for LH24n-B95) is even somewhat closer to the reference curve than the x-LMF-based one. As our initial training of the n-LMF (see discussion in Section 2) also included the EXX energy density as an input feature, we first thought that a similar mechanism may be operative as laid out in ref. (24) for the x-LMF. However, the n-LMF used finally in LH24n-B95 exhibits only meta-GGA-like input features and nevertheless suppresses the gauge problem successfully without a CF. This suggests an overall mechanism independent of whether the LMF includes the EXX energy density or not, calling for closer analysis.

**Figure 5:**
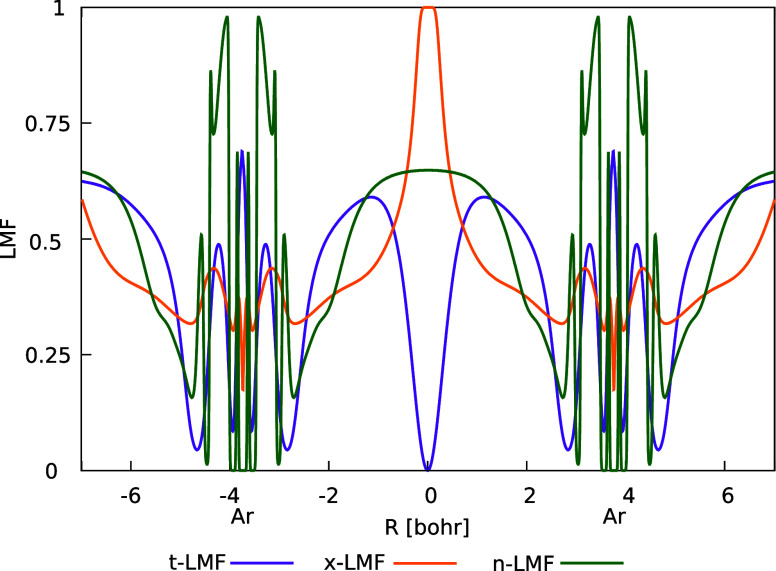
Comparison of the shape of t-LMF, x-LMF, and n-LMF for Ar_2_ at an internuclear distance of 7.5 bohr.

We can provide this by comparing LMF plots for such systems like Ar_2_. Figure 5 does this for an Ar–Ar distance of 7.5 bohr, where LH20t without CF exhibits substantial unphysical Pauli repulsion. As discussed above for a covalent bond, also in this case of absent bonding (without dispersion corrections) the t-LMF goes to zero at the BCP in the middle. That is, in this region of small densities between the two Ar atoms, the middle term is dominated entirely by the semilocal exchange-energy density, which is insufficiently negative. An unphysical positive integrand of the middle term of eq 1 results in this area between the atoms. In contrast, the x-LMF features a narrow but large peak with full EXX at the BCP. This should suppress unphysical Pauli repulsion in this area, consistent with a dramatic reduction of the gauge problem without a CF.^24^ The behavior of the n-LMF is yet a different one, with a broader but not quite as high peak around the BCP. Indeed the peak has a relatively flat plateau. Note that a GH does not suffer from a gauge problem, and a constant region may thus also be favorable for its reduction, in addition to the relatively large EXX admixture (we note that such broad maxima also start to develop upon stretching a covalent bond, as shown for the N_2_ molecule in Figure S3). It appears that the main problem of the t-LMF (and of other LMFs such as typical s-LMFs) in this context, which can be mitigated by adding a CF, is the observed zero and small EXX admixture at and near the BCP. We emphasize that in view of our previous analyses for the x-LMF^24^ we do not expect that the n-LMF eliminates all gauge-related problems, in the sense of making the integrand of the NDC term negative everywhere.^24^ However, it appears that the most detrimental aspects regarding NCIs can indeed be successfully suppressed without a CF. We will see the importance of this fact for NCI-related data further below when evaluating the GMTKN55 results. We should also note that these features of the n-LMF may have arisen in a somewhat coincidental way, as the training data do not cover NCIs to any notable extent. We finally mention that both the LH24x and LH24n-B95 Ar_2_ curves, as well as the underlying HF + B95c reference curve (Figure 4), are not completely “dispersionless” as one might expect them to be. This reflects the character of the B95c DC functional. However, the minima are exceedingly shallow in any case.

### 4.4 Evaluation of LH24n-B95 for the GMTKN55 Test Suite

As a primary evaluation database far beyond the training data we chose the widely used, large GMTKN55 test suite of general main-group thermochemistry, kinetics and noncovalent interactions.^52^Table 2 shows the WTMAD-2 values of the full suite, as well as for the usual five subcategories of “basic & small”, “iso & large”, “barriers”, “intermolecular NCIs” and “intramolecular NCIs”.^52^ We compare LH24n-B95-D4 to some of the known best-performing, parametrized rung 4 and very few rung 5 functionals having dispersion terms. The LH24t-D4 and LH24n-D4 results will be discussed further below.

**Table 2:** WTMAD-2 Values in kcal/mol for the Full GMTKN55 Database and Its Usual Subcategories, Comparison with the Best Literature Rung 4 and Rung 5 Functionals Including Dispersion Terms

	basic & small	iso & large	barriers	intermol. NCIs	intramol. NCIs	GMTKN55
LH20t-D4^25^	3.11	6.13	4.42	4.94	5.27	4.55^a^
LH24n-B95-D4^a^	2.33	5.33	3.04	3.60	4.02	3.49
LH24n-D4^a^	2.37	4.44	2.86	3.41	3.02	3.10
LH24t-D4^a^	3.16	4.55	3.92	3.52	4.94	3.90
ωLH23tdE-D4^21^	2.22	3.87	2.86	4.71	5.78	3.76^21^
DM21 (D3-BJ)^40^	1.99	4.64	3.63	6.56	4.16	3.97^40^
ωB97M-V^26^	2.73	4.79	3.40	2.90	4.53	3.53^104^
CF22D^28^	2.53	4.01	3.46	4.22	4.68	3.64^28^
B22plus,^27^^b^	2.33	3.50	2.83	2.12	3.43	2.79^27^
**rung 5**						
DSD-BLYP-D3(BJ)^105^	1.84	4.30	3.04	3.92	3.15	3.07^52^
ωB97M(2)^106^	1.39	2.61	1.66	2.44	3.00	2.13^107^

a This work.

b Nonself-consistent results based on BHandHLYP orbitals on 54 out of 55 subsets.

Strikingly, just replacing the t-LMF in LH20t-D4 by the n-LMF and reparameterizing the D4 correction to get LH24n-B95-D4 lowers the overall WTMAD-2 by more than 1 kcal/mol. This highlights the suboptimal nature of the t-LMF. The improvements are consistent across all subcategories, with reductions of more than 1.2 kcal/mol observed in barrier heights, inter- and intramolecular NCIs, and somewhat less for basic & small and iso & large. The overall WTMAD-2 value is already 0.3 kcal/mol lower than for our so far best RSLH, ωLH23tdE,^21^ which includes strong-correlation and delocalization-error corrections (see above), and 0.5 kcal/mol below the extensively trained NN functional DM21-D3(BJ),^40^ which should also be considered a strong-correlation-corrected RSLH. LH24n-B95-D4 is on par with the ωB97M-V^26^ RSH (with a VV10^108^ van-der-Waals-functional contribution), which for a long time was considered the best-performing rung 4 functional for this test suite.^104^ Notably, LH24n-B95-D4 has not been parametrized for GMTKN55 itself, albeit the choice of the LH20t-based B95c parameters helps in its performance. Strikingly, this good performance of LH24n-B95-D4 is obtained without a CF! We also include data for CF22D, which is a recent highly parametrized GH trained on large databases, and B22plus, which combines GH, sophisticated B05 features, and the Becke/Johnson dispersion model. B22plus, which is currently considered to give the lowest overall WTMAD-2 value of any rung 4 functional, has been essentially trained for GMTKN55, but has been used non-self-consistently only so far. We note in passing that of the functionals listed in Table 2, only ωLH23tdE and DM21 escape to some extent the zero-sum game between a minimization of delocalization and strong-correlation errors.^23^ While we have so far not introduced strong-correlation corrections into the n-LMF, work in that direction is ongoing.

We note in passing that our initial computational experiments with an n-LMF having hyper-meta-GGA input features, where we evaluated the full GMTKN55 suite every 1000 epochs up to 8000 epochs (see Figure S5), provided no improvement in WTMAD-2 beyond 2000 epochs, even though the data for the W4-17/BH76 optimization sets still varied. This indicates that the choice of a specific solution on the plateau of Figure 2 for the n-LMF may have only a minor impact on the wider overall quality of the functional.

## 5 Importance of the DC Functional, Arriving at LH24n

We have previously found that the parametrization of the B95c DC contribution has an appreciable influence on how the subsequent addition of dispersion contributions affects the description of NCIs, even if performance for small-molecule atomization energies or barriers may be affected very little. This suggested to us that the B95c formulation may not yet be sufficiently flexible. Therefore, we consider now the B97c-type power series expansion described in the Theory section above. The excellent performance of B22plus,^27^ which features among other contributions a sophisticated B98c^109^ DC term, is another hint that an improvement of the DC part could be relevant.

**Figure 6:**
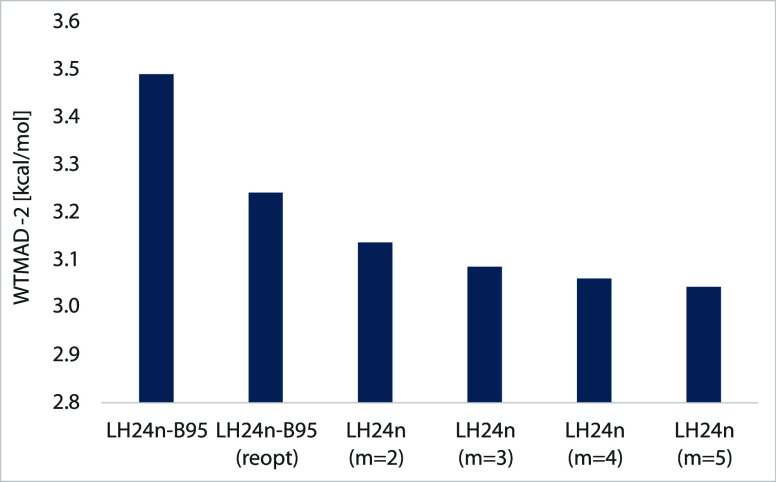
Post-SCF WTMAD-2 values for the full GMTKN55 set obtained with different DC forms, including a reoptimized B95c and different expansion lengths for the B97c-type DC functional.

While the n-LMF has been trained on the W4–17 and BH76 small-molecule sets, which do not feature much NCI contributions, it seemed reasonable to optimize the B97c-type DC terms on a larger database that also features such NCIs. We decided to use the full GMTKN55 WTMAD-2 value as a target, as has become rather common in recent years (see, e.g., refs. (110 and 111)). In addition to the linear parameters within the B97c-type power series (see above) we also optimized a linear scaling factor in front of the n-LMF to allow an adjustment to the altered DC contribution compared to LH24n-B95. As dispersion corrections are obviously crucial for the NCI subcategories, as well as for several subsets of the iso & large subcategory, the optimization needed to be done in the presence of dispersion contributions. We therefore performed it with the unaltered D4 contributions from LH24n-B95-D4. Indeed, initially we applied this procedure to the linear parameters *d*_opp_ and *d*_σσ_ (and the scaling factor of the n-LMF) in the B95c contribution of LH24n-B95-D4. This lowered the post-SCF WTMAD-2 (with LH24n-B95 orbitals) by 0.25 to 3.24 kcal/mol (see Figure 6). The n-LMF scaling factor became about 1.1, suggesting that slightly more overall EXX admixture is required for optimum WTMAD-2 in this setting than obtained during the previous LMF-training on W4-17 and BH76.

We then extended this procedure to the B97c-type formulation of the DC contribution, starting from a short expansion of the power series for both same-spin and opposite spin terms (*m* = 2, see Section 2) and increasing the series length stepwise up to *m* = 5 (Figure 6). We recall (see Section 2) that B95c can be interpreted as an *m* = 2 expansion after removing a mixed-power term in the same-spin contribution. Notable reductions in the post-SCF WTMAD-2 values are still seen from the reoptimized B95c to *m* = 2 and from *m* = 2 to *m* = 3. While some small further reductions occur at longer expansions, we decided to choose *m* = 3 as a reasonable compromise between performance and simplicity. This provides the final LH24n functional, and self-consistent data for LH24n-D4 are included in Table 2. The overall WTMAD-2 of 3.10 kcal/mol is the lowest self-consistent value of any rung 4 functional so far (as discussed above, the still somewhat lower B22plus value has been obtained without self-consistency). Of course double hybrids can achieve still lower values (Table 2), as GMTKN55 appears to be particularly well-suited for applying perturbational correlation contributions. We note furthermore that LH24n-D4 still falls somewhat behind our ωLH23tdE-D4 RSLH (and behind B22plus) for the iso & large subcategory. As ωLH23tdE-D4 utilizes 100% long-range EXX admixture and features both delocalization-error and strong-correlation-error corrections in the LMF, we expect that an extension to an RSLH framework may provide further improvements. This work is in progress. For comparison, we also replaced the B95c contribution in LH20t by a reoptimized B97c expansion (up to *m* = 5, including a mixed-power term), providing LH24t. Remarkably, LH24t-D4 (with D4 terms taken from LH20t-D4) reduces the overall WTMAD-2 by 0.65 kcal/mol relative to LH20t-D4 (Table 2), showing that the B97c-type series expansion offers notable improvements over B95c in the presence of both n- and t-LMFs. The improvements for LH24t-D4 arise from the NCI and iso and large subcategories but also from barriers, while basic & small is essentially unaffected.

As dispersion terms are important for NCIs, but also for intramolecular NCIs included in the iso & large subcategory of larger molecules, it is instructive to compare the results of the new functionals with and without the D4 contributions (Table 3). This holds even more so, as such atom-additive correction terms are not part of the underlying XC functional proper, even though such terms are sometimes optimized together with the functional. Of course this is more justified when using a true van-der-Waals functional, such as the VV10 contribution in the case of ωB97M-V. Table 3 shows that before addition of D4 corrections the two new functionals based on the n-LMF feature somewhat larger deviations for NCIs and iso and large than LH20t but exhibit an even larger reduction upon adding the D4 corrections. While this might arise from residual underlying gauge problems, we see no indications for this in the noble-gas dimer curves (e.g., in Figure 4). The improvements due to the D4 terms furthermore tend to be of similar magnitude as for many GHs, where the gauge problem is absent. Finally, earlier LHs without CF, which do exhibit significant gauge problems, show deviations for the NCI subcategories on the order of 20–40 kcal/mol.^25^ We therefore feel that the observed numbers fall within the usual variation width of the more or less “dispersionless” character of other functionals.

**Table 3:** Effects of D4 Corrections on the WTMAD-2 Values for GMTKN55 and Subcategories for a Number of LHs (Self-Consistent Results)

	basis & small	iso & large	barriers	intermol. NCIs	intramol. NCIs	GMTKN55
LH20t	3.13	8.50	4.03	11.20	12.63	7.58
LH20t-D4	3.11	6.13	4.42	4.94	5.27	4.55
LH24n-B95	2.55	10.15	3.27	17.18	15.72	9.37
LH24n-B95-D4	2.33	5.33	3.04	3.60	4.02	3.49
LH24n	2.67	9.19	3.86	17.96	16.87	9.71
LH24n-D4	2.37	4.44	2.86	3.41	3.02	3.10

The improvement of the barriers by ca. 1 kcal/mol for LH24n-D4 by the inclusion of the DFT-D4 terms was initially surprising. Closer analysis showed that this arises almost exclusively from the BHPERI subset (improvement by 2.4 kcal/mol), with a much smaller effect (ca. 0.66 kcal/mol) coming from both BHDIV10 and PX13 (see Table S4). BHPERI is indeed known for its sensitivity to dispersion corrections,^112,113^ and our MSE comparison of LH24n and LH24n-D4 (−3.37 kcal/mol) aligns with shifts observed for B3LYP-D3(BJ)^52^ (−4.58 kcal/mol) or PBE-D3(BJ)^52^ (−2.73 kcal/mol). For LH20t-D4 the MSE shift was −1.63 kcal/mol. In this case the MAE is almost unchanged, due to a compensation between changing negative and positive deviations. For BHDIV10 and PX13 the MSE shifts due to D4 corrections for LH24n are ca. 0.66 kcal/mol (coinciding with the negative of the MAE shift) compared to 0.35 and 0.23 kcal/mol, respectively, for LH20t-D4. Again, such shifts are similar to values obtained for many GHs.

### 5.1 Evaluation of the New Functionals for Organometallic Transition-Metal Reaction Energies and Barriers

As a yet more demanding test of the transferability of the n-LMF we also applied LH24n-B95-D4 and LH24n-D4 to a series of test sets representing organometallic transition-metal reactions. This includes the closed-shell reaction energy set MOR41,^84^ the open-shell reaction-energy set ROST61,^85^ and the MOBH28 barrier set^90^ (see Section 3). The detailed results in comparison with LH20t-D4 are provided in Tables S5–S8, while comparisons with other LHs and further functionals can be found in ref. (90). The MOBH28 barrier MAEs for LH24n-B95-D4 (1.74 kcal/mol) and LH24n-D4 (1.87 kcal/mol) are almost identical to that for LH20t-D4 (1.76 kcal/mol), close to the top-performing functionals of ref. (90). In contrast, the MAEs for MOR41 (LH24n-B95-D4 3.30 kcal/mol, LH24n-D4 3.65 kcal/mol) fall somewhat behind the LH20t-D4 value (2.25 kcal/mol). Similarly, we see the ROST61 MAEs (LH24n-B95-D4 3.38 kcal/mol, LH24n-D4 3.87 kcal/mol) to be larger than that of LH20t-D4 (2.44 kcal/mol), albeit also still in the range of well-performing functionals.^90^ These results indicate limitations in the transferability of the n-LMF performance to systems with rather different bonding situations. For a data-driven approach the obvious conceivable remedy is training of the LMF on a larger, more diverse test set including transition-metal systems. This may then require also a somewhat deeper and/or wider NN than the extremely small one used in this initial work, to represent the larger database. Alternatively, one may consider to add information on electron or energy densities to the training data.^77^ Work along these lines is in progress.

## 6 Conclusions

Local hybrid functionals based on a local admixture of semilocal and exact exchange-energy densities, and more sophisticated extensions derived from LHs, offer promising pathways to improve the accuracy of contemporary KS-DFT in various ways alluded to in the introduction. Here we have addressed improvements of two crucial aspects of LHs, (a) the local mixing function (LMF) that determines the position-dependence of EXX admixture, and (b) the dynamical correlation (DC) functional that complements the nondynamical correlation (NDC) middle term controlled by the LMF.

While many different forms of LMFs have been proposed in the literature, and some exact physical constraints are known for LMFs, most of the latter only pertain to the high-density or asymptotic limits and do not provide general guidelines to the shape of an LMF in the valence region that is most important for chemistry. Here we have therefore used a data-driven approach, i.e., we have constructed the LMF as a relatively shallow neural network (n-LMF). Replacing only the t-LMF in our previous successful LH20t functional by such an n-LMF provides already a striking improvement of both reaction barriers and atomization energies in the small data sets used for training the LMF, and also for the much larger GMTKN55 database used for further evaluations.

Replacing the B95c DC functional by a more flexible B97c-type power-series expansion and optimizing the parameters in the presence of dispersion corrections on data that also cover noncovalent interactions provides a smaller but still important improvement of the functional when applied to the GMTKN55 database. Combining the new n-LMF with such more flexible DC contributions in a balanced way, we arrive at the LH24n functional. LH24n-D4 provides the lowest deviations of an LH so far for the large GMTKN55 test suite, and the lowest WTMAD-2 value for any rung 4 functional so far in self-consistent calculations. Evaluation for organometallic transition-metal thermochemistry and reaction barriers reveals reasonable but less convincing performance. This suggests pathways for further improvements, e.g., more diverse training sets and larger neural networks.

The recent DM21 functional has received substantial attention in the DFT development community, as it demonstrated that extensively data-driven approaches can provide an important way to incorporate also important physical limits, such as a simultaneous reduction of delocalization errors and static correlation errors. The main disadvantage of such a deep neural-network functional is its complete black-box character. Here we have applied a much smaller neural network in a much more tailored way to a flexible but well-defined human-designed functional form. Only the LMF has been trained by a neural network. As the LMF is a simple real-space function with a small value range determining the EXX admixture of an LH, graphical evaluations for different molecules provide important insights into the LMF properties required for accurate thermochemistry and reaction barriers. For example, we find that the presence or absence of unphysical repulsive NDC contributions to noncovalent interactions seems to be related to the behavior of the LMF in the small-density region between the weakly interacting atoms. t-LMFs, which have been the most widely used ones so far, go to small values in such regions and thereby promote undesirable artifacts that have to be removed by a suitable calibration function. The new n-LMF and the recently proposed x-LMF of LH24x exhibit different behaviors in such regions. This can now be linked to the successful suppression of gauge-related artifacts by these LMFs. We note that, while the recent x-LMF had been constructed specifically to reduce the gauge problem, the n-LMF shares the favorable behavior even though the current training data do not contain any obvious connection to the gauge problem.

The n-LMF trained on atomization energies and reaction barriers also shows unexpectedly large EXX admixtures in covalent bonds. This suggests that large admixtures are quite acceptable when aiming not only for good barriers but also for good reaction energies. So far neither the core nor the asymptotic region of the LMF have been covered by the training data. One may expect improvements in these areas when addressing relevant properties in the optimization as well. Many further improvements are conceivable when extending the new approaches reported here to range-separated LHs and strong-correlation-corrected schemes.

## Supporting Information Available

The effects of the neural-network hyperparameters, optimized parameters of the functionals, LMF plots for linear diatomic molecules, correlation between training procedures and final performance, detailed results for GMTKN55, MOR41, ROST61 and MOBH28 test sets; algorithm describing loading weights and biases from file (PDF)
