# Supplementary material for: Data-Driven Improvement of Local Hybrid Functionals: Neural-Network-Based Local Mixing Functions and Power-Series Correlation Functionals
Source: J Chem Theory Comput. 2025 Jan 13;21(2):762–75. doi: 10.1021/acs.jctc.4c01503 (PMC11780747; doi:10.1021/acs.jctc.4c01503)
Supplement: Supplementary file 1 — ct4c01503_si_001.pdf [file ct4c01503_si_001.pdf]

Supplementary Information: Data-Driven Improvement of Local Hybrid Functionals: Neural-Network-Based Local Mixing Functions and Power-Series Correlation Functionals

Artur Wodyński,* Kilian Glodny, and Martin Kaupp*

Technische Universität Berlin, Institut für Chemie, Theoretische Chemie/Quantenchemie, Sekr. C7, Straße des 17. Juni 135, D-10623, Berlin, Germany

E-mail: artur.wodynski@tu-berlin.de; martin.kaupp@tu-berlin.de

Table S1. Self-consistent WTMAD-2 values for the GMTKN55 set and subcategories obtained with n-LMFs during testing with Becke's original B95c parameters. Only a selected subset of NN-based functionals has been assessed, with results reported solely for those yielding the lowest WTMAD-2 values when combined with D4 dispersion corrections. The features applied in the n-LMF are of the hyper-meta-GGA set. The hyperparameters are presented in the format [number of nodes per layer] × [number of layers], [activation function]

hyperparameters	basic & small	iso & large	barrier heights	inter NCIs	intra NCIs	total
64 × 3, gelu	2.41	7.09	3.05	9.43	8.86	5.91
64 × 3, gelu, D4	2.33	5.58	2.98	5.09	9.20	4.82
128 × 4, gelu	2.39	5.43	2.93	7.87	8.18	5.18
128 × 4, gelu, D4	2.34	4.45	3.00	4.95	9.12	4.60
64 × 3, tanh	2.65	6.58	2.74	8.30	7.88	5.45
64 × 3, tanh, D4	2.57	5.41	2.83	5.20	9.13	4.86
128 × 4, tanh	3.04	7.27	3.49	7.45	8.97	5.82
128 × 4, tanh, D4	2.99	6.37	3.45	5.67	9.74	5.44

Table S2. Self-consistent WTMAD-2 values for the GMTKN55 set and subcategories obtained with n-LMFs during testing with the B95c parameters from LH20t. Only a selected subset of NN-based functionals has been assessed, with results reported solely for those yielding the lowest WTMAD-2 values when combined with D4 dispersion corrections. The features applied in the n-LMF are of the hyper-meta-GGA set. The hyperparameters are presented in the format [number of nodes per layer] × [number of layers], [activation function]

hyperparameters	basic & small	iso & large	barrier heights	inter NCIs	intra NCIs	total
64 × 3, gelu	2.66	9.62	3.25	17.27	14.89	9.18
64 × 3, gelu, D4	2.29	4.98	3.18	3.77	4.29	3.52
128 × 4, gelu	2.63	8.46	2.87	16.37	15.06	8.78
128 × 4, gelu, D4	2.50	4.25	3.60	4.87	6.54	4.19
64 × 3, tanh	2.63	9.42	2.55	16.55	14.81	8.88
64 × 3, tanh, D4	2.28	4.72	2.78	3.80	4.34	3.44
128 × 4, tanh	2.80	9.44	3.63	16.56	13.90	8.90
128 × 4, tanh, D4	2.55	5.06	3.27	4.40	5.18	3.93

Table S3. Parameterization of B95c/B97c correlation functionals and D4 dispersion corrections, and additional scaling factors, for LH24n-D4, LH24n-B95-D4, and LH24t-D4

		LH24n-B95-D4[a]	LH24n-D4[b]	LH24t-D4[c]
	n-LMF/t-LMF scaling factor	1.0000	1.0996	0.696
	nlx	1.0	1.0	0.781
B95c/B97c	c_{opp}	0.004987	0.004987	0.004987
	$c_{\sigma\sigma}$	0.09544	0.09544	0.09544
	d_{opp} (B95c)	1.2291	-	-
	$d_{opp,0}$ (B97c)	-	1.40138769	1.3588727
	$d_{opp,1}$ (B97c)	-	-2.90212804	-4.01387823
	$d_{opp,2}$ (B97c)	-	3.41403089	7.8988689
	$d_{opp,3}$ (B97c)	-	-1.83609439	-4.00083644
	$d_{opp,4}$ (B97c)	-	-	-7.20419559
	$d_{opp,5}$ (B97c)	-	-	9.13339205
	$d_{\sigma\sigma}$ (B95c)	0.8178	-	
	$d_{\sigma\sigma,0}$ (B97c)	-	0.22875368	-0.018561
	$d_{\sigma\sigma,1}$ (B97c)	-	-0.52818435	1.39162825
	$d_{\sigma\sigma,2}$ (B97c)	-	0.91060473	-5.64989112
	$d_{\sigma\sigma,3}$ (B97c)	-	-0.6228437	4.89534155
	$d_{\sigma\sigma,4}$ (B97c)	-	-	2.93225131
	$d_{\sigma\sigma,5}$ (B97c)	-	-	-3.5537405
	$d_{\sigma\sigma,mix}$ (B97c)	-	-	1.39139694
D4	s8	0.16091	0.16091	0.113
	a1	0.46224	0.46224	0.479
	a2	3.67510	3.67510	4.635

Footnotes to Table S3

[a]Functional can be called with the LH24n-B95 keyword in Turbomole 7.9.

[b]Keyword for the functional is not yet implemented in Turbomole 7.9. Please use:

```
$dft
 functional local-hybrid
 hybparams x=pbe c=b97 nlx=1.0 nlc=1.0
 lmf n-lmf1 1.09963514
 b97-c 0.09544 0.004987 0.22875368 -0.52818435  0.91060473 -0.6228437 0.0 0.0 0.0 1.40138769 -2.90212804  3.41403089 -1.83609439 0.0 0.0
 gauge none
```

[c]Keyword for the functional is not yet implemented in Turbomole 7.9. Please use:

```
$dft
 functional local-hybrid
 hybparams x=pbe c=b97 nlx=0.781 nlc=1.0
 lmf common t-lmf 0.696
 b97-c 0.09544 0.004987 -0.018561 1.39162825 -5.64989112  4.89534155  2.93225131 -3.5537405   1.39139694 1.3588727 -4.01387823  7.8988689  -4.00083644 -7.20419559  9.13339205
 gauge pig2 -0.936124 0.203433 becke 0.004936
```

Algorithm 1 Loading Weights and Biases from File (https://doi.org/10.5281/zenodo.13969761)

```
Initialize num_layers = 3 , num_neurons_layer = 64, and num_features = 7
Initialize an array for weights: weights[num_layers, num_neurons, num_neurons_layer]
Initialize an array for biases: biases[num_layers, num_neurons_layer]
Open file for reading
for each layer L from 1 to num_layers do
    if L is 1 (first layer) then
        Set num_inputs = num_features
    else
        Set num_inputs = num_neurons_layer
    end if
    if L is the 3 (last layer) then
        Set num_neurons = 1
    else
        Set num_neurons = num_neurons_layer
    end if
    for each neuron N in layer L do
        for each input I to neuron N do
            Read weight from file and store in weights[L, N, I]
        end for
        Read bias for neuron N and store in biases[L, N]
    end for
end for
Close file
```

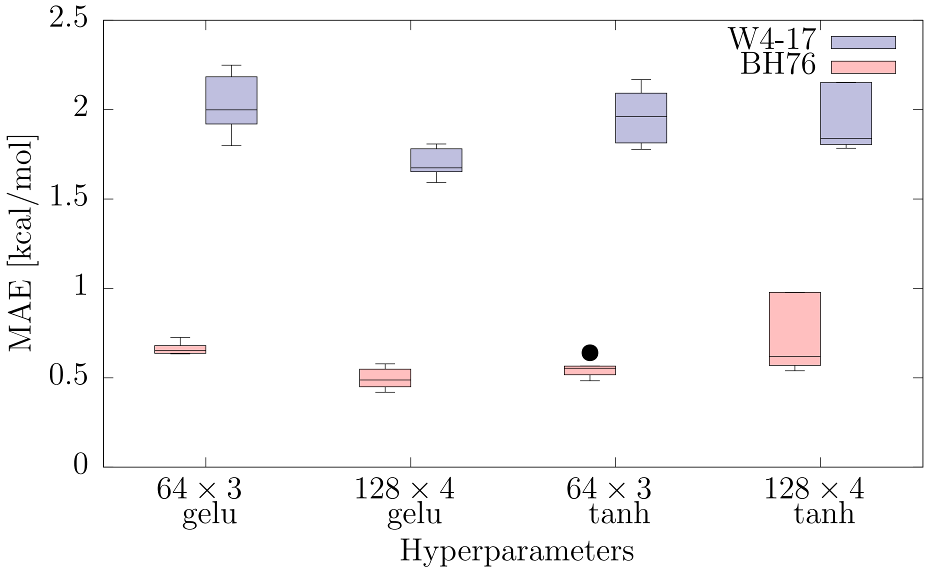

Figure S1. MAE of the SCF results for the hyper-meta-GGA set of input features for n-LMFs. The B95c parameters from LH20t were used. Additional outliers were observed for the hyperparameters 128×4, tanh (4.5 kcal/mol and 8.5 kcal/mol for the BH76 and W4-17 test sets, respectively). Each hyperparameter set corresponds to six independently trained NN functionals (three different seeds for random initialization, each trained twice). Only the version of each NN functional that achieves the lowest MAE, when evaluated self-consistently over the training data during the epoch scan, is considered.

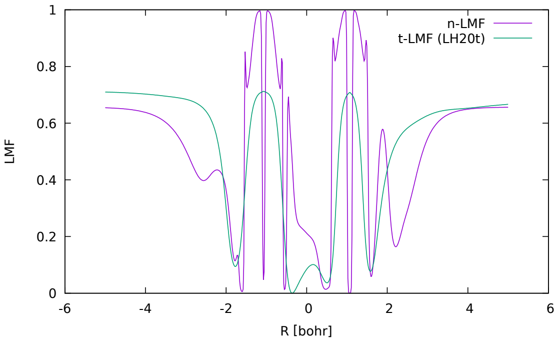

(a) CO

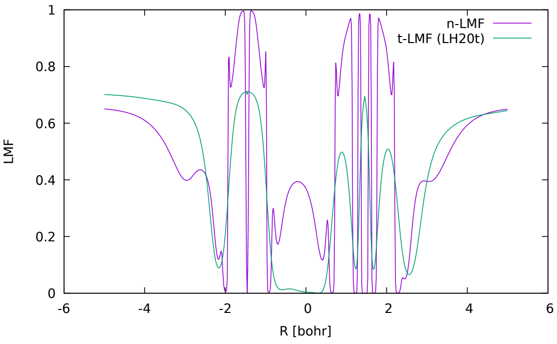

(b) CS

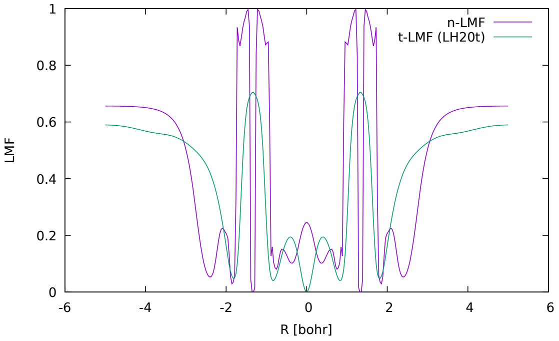

(c) F_2

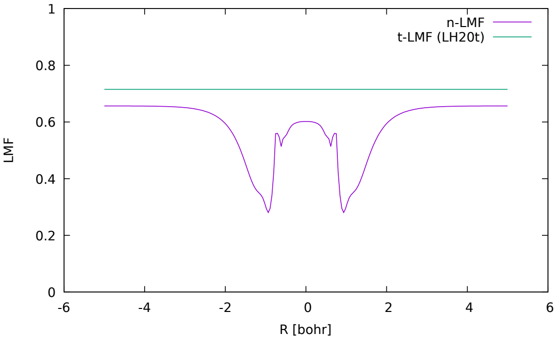

(d) H_2

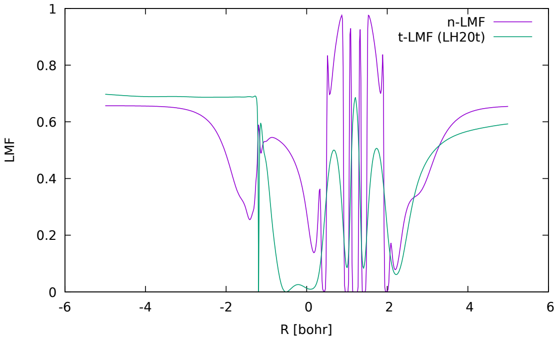

(e) HCl

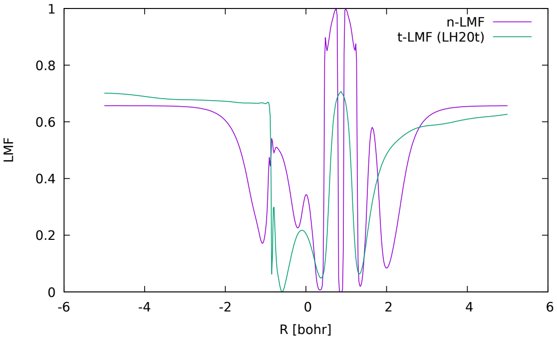

(f) HF

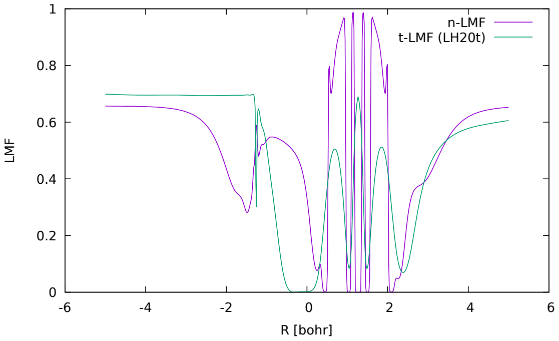

(g) HS

Figure S2. Plot of final n-LMF of LH24n-B95 for selected diatomic molecules along the bond axis (magenta) compared to the scaled t-LMF of LH20t (green). (a) CO, (b) CS, (c) F_2, (d) H_2, (e) HCl, (f) HF, and (g) HS.

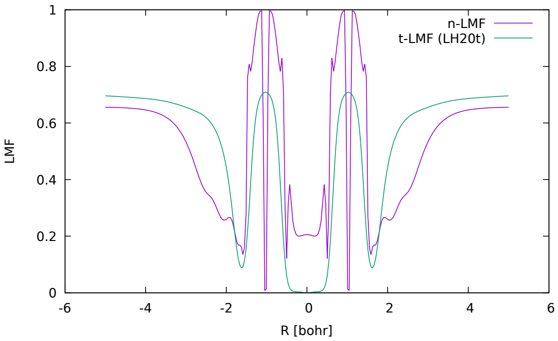

(a) N_2, ΔR = 0.0 bohr

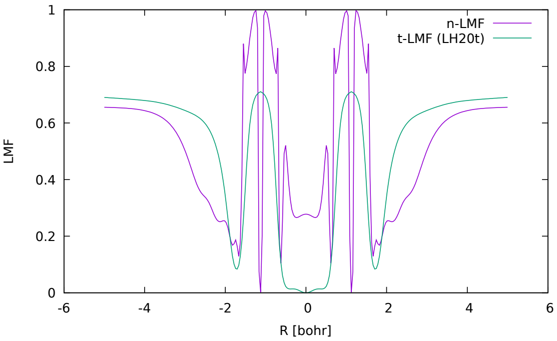

(b) N_2, ΔR = 0.2 bohr

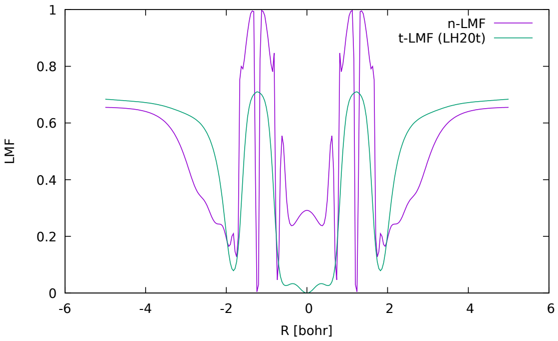

(c) N_2, ΔR = 0.4 bohr

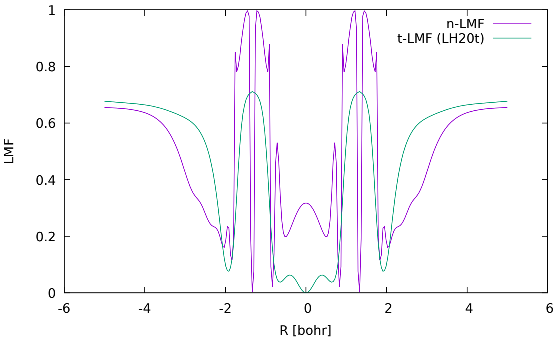

(d) N_2, ΔR = 0.6 bohr

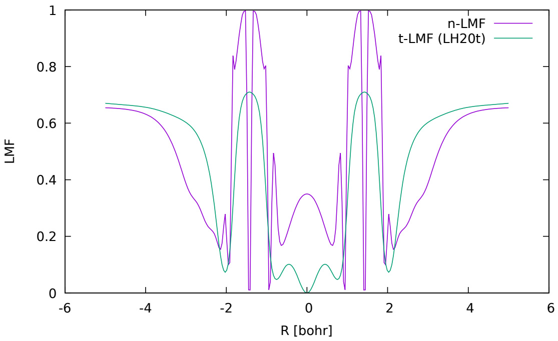

(e) N_2, ΔR = 0.8 bohr

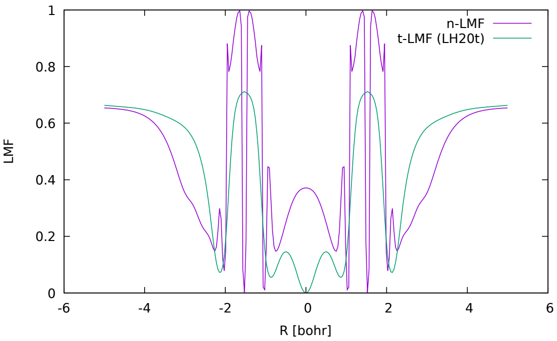

(f) N_2, ΔR = 1.0 bohr

Figure S3. Plot of final n-LMF of LH24n-B95 along the bond axis (magenta) compared to the scaled t-LMF of LH20t (green) for N_2 at different (stretched) bond lengths (ΔR = 0.0, +0.2, +0.4, +0.6, +0.8, +1.0 bohr). ΔR indicates the deviation from the equilibrium bond length.

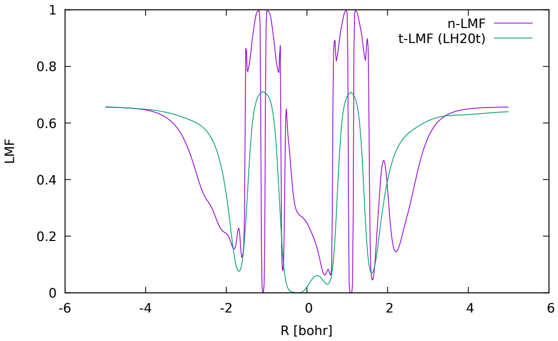

(a) NO, $\Delta R = 0.0$ bohr

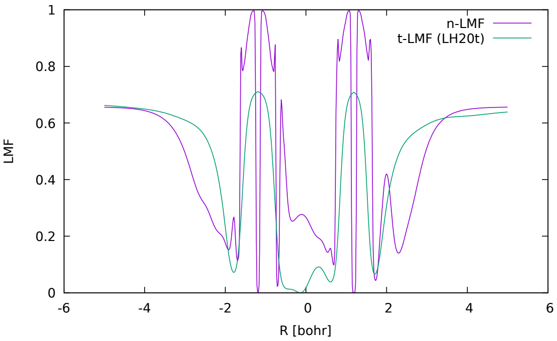

(b) NO, $\Delta R = 0.2$ bohr

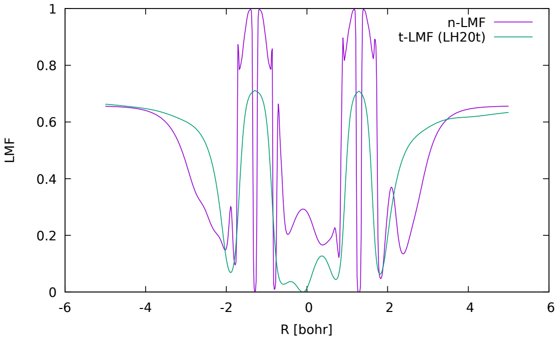

(c) NO, $\Delta R = 0.4$ bohr

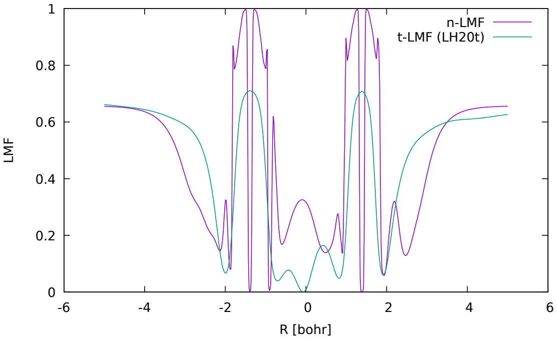

(d) NO, $\Delta R = 0.6$ bohr

Figure S4. Plot of final n-LMF of LH24n-B95 along the bond axis (magenta) compared to the scaled t-LMF of LH20t (green) for NO at different (stretched) bond lengths ($\Delta R = 0.0$, +0.2, +0.4, +0.6 bohr). ΔR indicates the deviation from the equilibrium bond length.

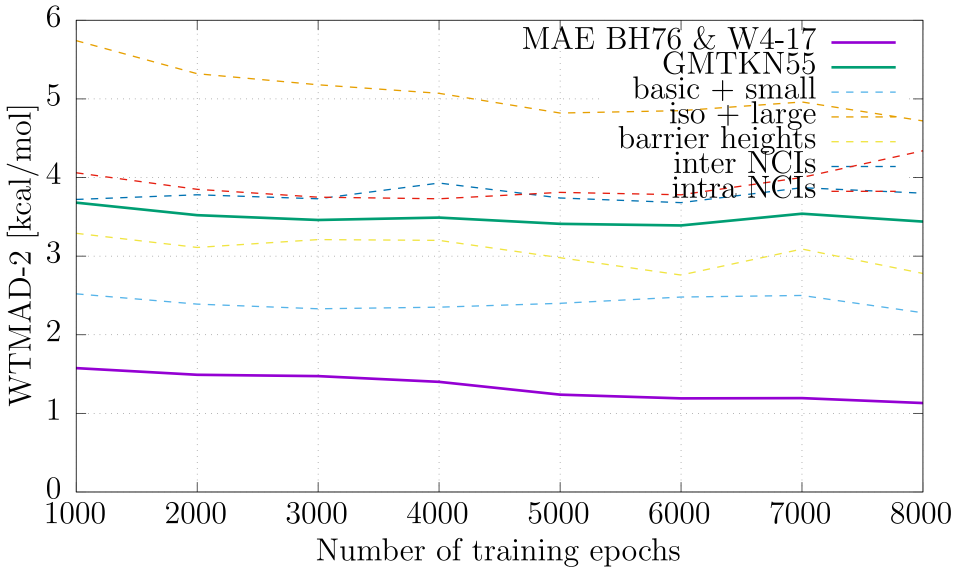

Figure S5. WTMAD-2 for the GMTKN55 set and its subcategories for an n-LMF with hyper-meta-GGA input features, 64 × 3 NN, tanh activation function and B95c parameters from LH20t extracted for every 1000 training epochs. Self-consistent results with reoptimized D4 dispersion corrections for a given n-LMF. The W4-17/BH76 MAE (without D4 corrections) used for training is also shown along the same epochs.

Table S4. Comparison of GMTKN55 subset results (in kcal/mol) for LH24n, LH24n-B95, LH20t and LH24t with and without D4 corrections

	LH24n-B95-D4		LH24n-B95		LH24n-D4		LH24n		LH20t-D4		LH20t		LH24t-D4		LH24t	
	MSD	MAD	MSD	MAD	MSD	MAD	MSD	MAD	MSD	MAD	MSD	MAD	MSD	MAD	MSD	MAD
ACONF	-0.16	0.16	0.49	0.49	-0.08	0.08	0.57	0.57	0.07	0.07	0.45	0.45	0.10	0.10	0.48	0.48
ADIM6	0.16	0.16	-3.05	3.05	0.12	0.12	-3.09	3.09	-0.06	0.14	-2.41	2.41	0.22	0.22	-2.12	2.12
AHB21	-0.09	0.21	0.75	0.80	0.01	0.25	0.86	0.88	-0.68	0.69	-0.26	0.57	-0.16	0.39	0.26	0.54
AL2X6	-0.50	0.53	-3.79	3.79	-1.07	1.07	-4.36	4.36	-1.26	1.26	-3.10	3.10	-2.29	2.29	-4.13	4.13
ALK8	0.62	0.86	-0.39	1.61	-1.05	1.53	-2.07	2.29	0.18	1.61	-0.42	2.00	-1.92	2.40	-2.52	2.85
ALKBDE10	-0.23	3.57	-0.65	3.62	-1.24	3.91	-1.67	4.02	0.15	3.32	-0.02	3.31	-2.14	3.94	-2.31	4.01
AMINO20x4	0.01	0.16	-0.07	0.37	-0.02	0.14	-0.09	0.43	0.17	0.25	0.14	0.32	0.11	0.26	0.08	0.35
BH76	-1.07	1.28	-0.45	0.91	-0.45	1.20	0.17	1.19	-1.26	2.21	-0.99	2.05	-1.58	2.04	-1.31	1.84
BH76RC	0.02	0.94	0.03	0.80	0.05	0.96	0.06	0.88	0.02	1.60	0.03	1.59	-0.18	1.78	-0.18	1.79
BHDIV10	-1.33	1.42	-0.43	1.08	-0.26	0.85	0.64	1.51	-1.28	1.43	-0.93	1.11	-0.85	1.43	-0.50	1.28
BHPERI	-0.39	1.28	2.98	3.13	-0.01	0.99	3.36	3.38	-0.89	1.48	0.74	1.04	-1.02	1.31	0.61	0.96
BHROT27	0.33	0.38	0.27	0.35	0.26	0.32	0.21	0.28	0.45	0.50	0.44	0.50	0.35	0.39	0.34	0.39
BSR36	-1.88	1.88	-7.20	7.20	-1.99	1.99	-7.31	7.31	-1.27	1.28	-4.42	4.42	-0.84	0.85	-4.00	4.00
BUT14DIOL	-0.05	0.08	-0.52	0.52	-0.06	0.07	-0.53	0.54	-0.07	0.12	-0.32	0.32	-0.17	0.18	-0.42	0.42
C60ISO	2.53	2.53	3.18	3.18	5.27	5.27	5.92	5.92	-1.45	1.70	-1.01	1.44	-1.67	1.84	-1.22	1.45
CARBHB12	0.57	0.57	-0.22	0.44	0.50	0.50	-0.29	0.46	0.68	0.70	0.21	0.53	0.40	0.50	-0.08	0.50
CDIE20	0.82	0.83	0.98	1.02	0.48	0.55	0.63	0.68	0.75	0.76	0.82	0.82	0.40	0.50	0.46	0.54
CHB6	0.10	0.54	0.13	0.64	0.16	0.55	0.19	0.69	-0.10	0.64	-0.06	0.55	-0.02	0.58	0.03	0.54
DARC	-0.64	1.20	5.13	5.13	-0.94	0.95	4.83	4.83	0.36	1.24	3.27	3.40	0.16	1.01	3.07	3.19
DC13	-0.83	5.38	0.44	7.43	-1.50	4.07	-0.23	6.11	-3.13	8.93	-2.49	8.58	-2.96	7.42	-2.32	6.87
DIPCS10	-3.45	4.05	-4.05	4.46	-5.37	6.13	-5.98	6.49	-5.92	6.65	-6.14	6.85	-7.22	7.76	-7.45	7.96
FH51	-0.21	1.44	0.85	1.76	-0.30	1.66	0.75	1.76	0.26	1.98	0.77	2.16	0.45	2.20	0.97	2.34
G21EA	0.54	1.33	0.22	1.48	-0.03	1.78	-0.35	1.95	-0.17	1.69	-0.28	1.77	-0.59	2.08	-0.71	2.16
G21IP	-0.23	2.57	-0.35	2.61	-0.71	3.25	-0.83	3.32	0.06	3.29	0.02	3.31	-0.62	3.78	-0.66	3.81
G2RC	-0.99	2.75	-0.53	2.16	-1.14	2.79	-0.68	2.10	-0.48	3.06	-0.31	2.86	0.05	2.92	0.22	2.64
HAL59	0.20	0.37	-1.05	1.10	0.12	0.35	-1.13	1.16	0.47	0.58	-0.27	0.78	0.27	0.43	-0.47	0.76
HEAVY28	-0.06	0.25	-0.72	0.72	-0.11	0.28	-0.76	0.76	0.13	0.21	-0.26	0.31	-0.01	0.17	-0.40	0.40
HEAVYSB11	-0.06	0.78	-2.30	2.39	0.23	0.97	-2.01	2.01	2.15	2.15	0.86	1.60	0.59	1.63	-0.70	1.72
ICONF	0.02	0.16	0.07	0.34	-0.01	0.18	0.04	0.36	0.13	0.31	0.15	0.37	0.04	0.31	0.06	0.36
IDISP	0.68	1.10	1.67	9.13	0.36	1.12	1.35	9.68	1.56	1.58	1.99	5.32	1.60	1.60	2.03	5.29
IL16	0.99	0.99	3.73	3.73	1.20	1.20	3.94	3.94	1.27	1.27	2.77	2.77	1.59	1.59	3.08	3.08
INV24	0.06	0.88	-0.53	1.07	0.41	1.06	-0.18	1.16	-0.32	1.12	-0.67	1.35	-0.15	0.98	-0.50	1.21
ISO34	-0.56	1.06	-0.74	1.36	-0.21	0.71	-0.40	1.03	-1.24	1.69	-1.33	1.80	-0.97	1.36	-1.06	1.47
ISOL24	-0.82	2.01	-2.76	4.59	-0.17	1.41	-2.11	4.02	-1.52	2.88	-2.44	4.38	-0.92	1.93	-1.84	3.49
MB1643	-6.68	10.95	-24.65	24.67	-17.36	20.25	-35.34	35.34	9.71	12.19	0.47	8.27	0.32	11.91	-8.92	13.59
MCONF	0.14	0.27	-1.31	1.38	0.03	0.11	-1.43	1.48	0.15	0.26	-0.91	0.93	0.14	0.29	-0.92	0.93
NBPRC	0.10	1.15	2.24	2.58	0.74	1.57	2.87	3.21	0.22	1.22	1.25	1.65	1.02	2.07	2.05	2.58
PA26	1.06	1.22	1.35	1.48	1.91	1.91	2.20	2.20	-0.47	1.38	-0.35	1.38	0.74	1.19	0.86	1.27
PArel	0.36	0.72	0.31	0.79	0.28	0.73	0.23	0.79	0.50	0.88	0.48	0.89	0.41	0.86	0.40	0.85
PCONF21	0.17	0.37	0.01	2.09	0.04	0.20	-0.12	2.15	0.30	0.60	0.16	2.02	0.26	0.34	0.11	1.71
PNICO23	0.28	0.34	-1.00	1.00	0.25	0.30	-1.03	1.03	0.47	0.47	-0.25	0.40	0.31	0.32	-0.41	0.47
PX13	0.60	0.90	1.30	1.34	1.16	1.21	1.86	1.86	-0.57	0.57	-0.34	0.50	-0.15	0.19	0.08	0.23
RC21	1.17	1.95	-0.46	1.26	0.72	1.62	-0.91	1.38	2.25	3.62	1.50	3.25	1.96	3.31	1.21	2.94
RG18	-0.01	0.07	-0.41	0.41	-0.02	0.06	-0.43	0.43	-0.10	0.15	-0.37	0.37	-0.01	0.08	-0.27	0.27
RSE43	-0.86	0.86	-1.00	1.00	-0.35	0.44	-0.50	0.58	-1.18	1.21	-1.25	1.28	-0.50	0.76	-0.58	0.79
S22	0.11	0.21	-2.56	2.56	-0.03	0.17	-2.70	2.70	0.23	0.28	-1.60	1.64	0.05	0.25	-1.78	1.78
S66	0.11	0.15	-2.13	2.13	0.03	0.12	-2.21	2.21	0.15	0.23	-1.41	1.42	0.05	0.14	-1.50	1.50
SCONF	0.03	0.30	-0.25	0.46	-0.08	0.15	-0.36	0.68	0.11	0.32	0.01	0.13	-0.12	0.14	-0.22	0.43
SIE4x4	8.67	8.67	8.44	8.44	6.90	6.90	6.67	6.67	7.44	7.44	7.32	7.32	8.14	8.14	8.03	8.03
TAUT15	-0.12	0.60	-0.16	0.57	-0.23	0.59	-0.27	0.58	0.26	1.06	0.27	1.08	0.04	0.80	0.05	0.81
UPU23	0.76	0.76	1.74	1.91	0.83	0.83	1.82	1.94	0.63	0.70	1.41	1.59	0.50	0.63	1.28	1.41
W411	0.32	3.16	-0.93	2.68	-2.79	3.75	-4.04	4.58	-0.03	2.99	-0.51	2.89	-4.32	5.06	-4.80	5.38
WATER27	1.02	1.11	-5.77	6.76	0.56	0.91	-6.24	7.21	2.42	2.53	-1.32	2.82	-0.84	2.17	-4.57	5.66
WCPT18	-0.07	1.13	0.85	0.96	0.97	1.60	1.89	1.89	-0.37	1.33	0.03	0.97	0.09	1.22	0.48	0.99
YBDE18	0.45	1.87	-2.32	3.11	-0.01	1.89	-2.78	3.23	2.26	2.31	0.97	1.79	0.32	1.13	-0.97	1.55

Table S5. Comparison of MOR41 reaction-energy results and statistical data (in kcal/mol) for LH24n-D4, LH24n-B95-D4, and LH20t-D4

Reaction	Reference	LH20t-D4	LH24n-B95-D4	LH24n-D4
1	-43.10	-42.30	-39.46	-38.11
2	-46.60	-44.12	-38.12	-36.65
3	-27.60	-23.41	-15.86	-14.23
4	-62.50	-64.08	-59.53	-61.06
5	3.70	-1.96	0.94	1.31
6	-23.20	-17.58	-18.07	-17.28
7	-16.20	-12.73	-15.01	-14.12
8	-17.20	-8.21	-12.30	-12.65
9	-18.70	-5.52	-10.74	-12.25
10	-22.60	-16.63	-13.99	-13.63
11	27.00	29.29	34.03	33.89
12	-29.80	-29.60	-27.02	-25.45
13	-43.20	-43.49	-40.58	-39.66
14	-52.00	-51.61	-43.67	-42.24
15	-4.10	-4.79	-13.24	-17.72
16	-39.80	-39.73	-40.10	-38.50
17	-16.10	-14.27	-15.61	-15.46
18	-34.20	-32.73	-31.10	-31.45
19	-40.10	-39.27	-37.42	-38.05
20	-30.20	-30.32	-29.34	-30.00
21	-15.10	-16.93	-17.65	-17.05
22	-35.90	-34.97	-30.73	-30.42
23	-55.00	-52.26	-49.45	-50.47
24	-41.60	-41.54	-40.55	-40.08
25	-45.90	-45.82	-44.85	-43.99
26	-36.40	-35.20	-36.71	-38.80
27	-21.80	-23.43	-23.97	-24.46
28	-36.30	-35.98	-37.16	-38.73
29	-28.30	-26.17	-28.71	-29.96
30	-14.00	-10.94	-15.09	-16.83
31	-29.90	-26.69	-28.49	-30.65
32	-1.80	-2.08	-0.56	-0.33
33	-10.70	-7.84	-10.13	-12.15
34	-25.60	-24.61	-23.73	-25.38
35	-30.90	-29.92	-29.05	-30.81
36	-39.80	-36.88	-34.57	-36.66
37	-14.00	-16.31	-16.24	-18.12
38	-64.40	-62.55	-63.15	-66.55
39	-63.90	-64.19	-60.99	-60.70
40	-65.80	-67.12	-66.31	-66.39
41	-3.20	-2.21	-2.07	-3.16

	LH20t-D4	LH24n-B95-D4	LH24n-D4
MAE	2.25	3.30	3.65
RMSD	3.44	4.44	5.01
MSE	1.47	2.21	1.75
MaxE(−)	-5.66	-9.14	-13.62
MaxE(+)	13.18	11.74	13.37
STDEV	3.11	3.85	4.68

Table S6. Comparison of ROST61 reaction-energy results and statistical data (in kcal/mol) for LH24n-D4, LH24n-B95-D4, and LH20t-D4

	ref	LH20t-D4	LH20t-D4	LH24n-B95-D4
1	-40.05	-25.83	-30.34	-29.74
2	-37.71	-48.68	-48.55	-46.61
3	-12.59	-12.59	-14.21	-14.25
4	-17.04	-16.62	-12.78	-14.44
5	-10.66	-10.62	-11.92	-12.24
6	-12.66	-6.84	-7.27	-6.27
7	-14.73	-15.55	-14.76	-15.34
8	-10.51	-9.16	-10.91	-10.20
9	-12.76	-10.58	-13.11	-12.63
10	-21.20	-25.10	-25.62	-26.05
11	-8.44	-14.51	-12.51	-13.42
12	-2.79	-1.70	-1.19	-1.27
13	-178.96	-190.21	-198.16	-195.97
14	-42.33	-45.07	-45.75	-44.83
15	-7.17	-8.49	-7.91	-7.35
16	-193.43	-192.03	-183.49	-186.49
17	-200.10	-200.35	-193.56	-196.11
18	-203.09	-202.61	-196.49	-198.84
19	-4.94	1.36	6.50	5.69
20	-27.02	-28.00	-28.75	-29.47
21	-25.09	-27.24	-29.42	-28.99
22	-46.70	-47.28	-47.54	-47.60
23	-115.35	-110.49	-109.42	-108.82
24	-46.37	-47.62	-48.61	-48.50
25	-19.39	-13.79	-11.87	-10.08
26	-47.73	-49.95	-49.55	-50.04
27	-25.86	-24.52	-23.58	-23.24
28	-28.77	-27.04	-26.75	-26.26
29	-11.13	-11.81	-11.77	-11.81
30	-141.32	-141.29	-142.18	-141.31
31	-63.73	-63.26	-58.11	-60.55
32	-49.75	-51.30	-49.74	-49.29
33	-10.64	-23.50	-20.47	-18.63
34	-0.65	-0.77	-0.67	-0.82
35	-5.46	-18.49	-15.12	-14.36
36	-49.04	-47.20	-42.27	-42.93
37	-40.62	-39.71	-39.66	-39.32
38	-27.70	-28.32	-37.73	-36.29
39	-44.99	-45.40	-49.02	-47.94
40	-24.51	-24.65	-23.00	-24.16
41	-5.96	-6.50	-6.46	-6.33
42	-26.96	-24.15	-25.54	-26.71
43	-80.58	-85.31	-85.07	-85.08
44	-66.15	-71.06	-70.43	-70.73
45	-42.83	-43.47	-47.56	-46.37
46	-33.27	-31.98	-31.09	-31.93
47	-58.47	-57.63	-55.91	-57.44
48	-4.24	-4.95	-8.72	-7.67
49	-35.26	-34.98	-34.27	-33.60
50	-2.74	-2.59	-0.50	-1.40
51	-29.77	-30.06	-34.39	-33.06
52	-42.72	-44.09	-49.55	-47.82
53	-30.78	-30.66	-30.34	-29.85
54	-12.33	-11.74	-7.89	-8.30
55	-4.61	-4.67	-4.59	-4.30
56	-13.65	-13.58	-14.00	-13.90
57	-31.89	-31.95	-28.45	-29.94
58	-39.30	-40.04	-38.00	-39.38
59	-29.89	-26.91	-30.12	-30.75
60	-66.00	-65.06	-61.53	-63.03
61	-69.48	-70.40	-71.11	-71.15

		LH20t-D4	LH20t-D4	LH24n-B95-D4
MAE		2.44	3.87	3.38
RMSD		4.21	5.35	4.75
MSE		-0.47	-0.21	-0.26
MaxE(–)		-13.03	-19.20	-17.01
MaxE(+)		14.22	11.44	10.63
STDEV		4.22	5.39	4.78

Table S7. Comparison of MOBH28 reaction-barrier results (in kcal/mol) for LH24n-D4, LH24n-B95-D4, and LH20t-D4

barrier	ref	LH20t-D4	LH24n-D4	LH24n-B95-D4
1_fwd	26.20	26.49	28.37	27.31
2_fwd	5.71	6.71	6.93	7.14
3_fwd	0.92	0.90	1.18	1.11
4_fwd	1.36	0.63	0.91	0.92
5_fwd	4.63	5.87	5.70	5.22
6_fwd	15.76	15.14	15.51	15.09
7_fwd	27.59	26.51	27.33	26.11
8_fwd	34.57	33.31	31.27	32.13
10_fwd	-4.29	0.86	-1.42	0.15
11_fwd	29.49	29.05	27.28	28.40
12_fwd	5.50	4.86	4.65	4.66
13_fwd	20.65	18.34	23.48	21.23
14_fwd	10.10	10.65	10.30	10.21
15_fwd	20.66	17.95	20.24	18.42
16_fwd	35.45	34.98	33.14	33.12
21_fwd	8.41	8.58	9.79	8.44
22_fwd	13.84	12.35	14.71	13.49
23_fwd	29.45	28.53	31.97	31.07
26_fwd	25.83	20.80	22.03	22.10
27_fwd	14.05	12.82	12.63	12.39
28_fwd	30.18	31.04	29.52	29.11
29_fwd	14.72	15.52	14.19	14.06
30_fwd	9.79	11.58	10.64	9.87
31_fwd	2.91	7.65	6.68	6.50
32_fwd	20.18	20.90	22.14	21.29
33_fwd	1.05	0.01	1.14	0.85
34_fwd	29.16	27.63	26.57	25.95
35_fwd	17.28	16.09	15.84	16.00
1_rev	14.02	14.70	18.05	16.00
2_rev	22.25	20.00	23.39	22.52
3_rev	26.92	32.83	32.00	31.41
4_rev	8.25	11.71	12.13	11.61
5_rev	22.60	22.01	21.85	21.69
6_rev	14.61	12.95	12.57	12.07
7_rev	18.58	16.35	18.42	17.47
8_rev	31.82	29.34	23.63	24.33
10_rev	8.22	2.40	6.40	5.91
11_rev	82.34	80.96	84.56	83.26
12_rev	37.18	40.16	38.19	37.08
13_rev	47.99	48.83	52.70	50.54
14_rev	14.37	13.83	15.26	14.45
15_rev	74.98	81.15	78.83	80.97
16_rev	53.77	57.41	57.12	58.23
21_rev	8.41	8.58	9.79	8.45
22_rev	27.01	25.09	28.58	25.84
23_rev	20.35	20.47	22.58	22.33
26_rev	0.11	0.07	0.14	0.20
27_rev	2.29	1.97	1.81	1.56
28_rev	15.52	15.93	15.87	15.56
29_rev	31.19	29.88	29.60	28.84
30_rev	16.60	17.91	19.39	18.49
31_rev	12.90	11.72	11.85	11.28
32_rev	62.62	69.39	63.83	67.48
33_rev	7.86	10.32	11.43	11.12
34_rev	3.04	4.88	5.39	4.98
35_rev	-2.44	-2.30	-2.08	-2.06

Table S8. Comparison of MOBH28 barrier-set statistical data (in kcal/mol) for LH24n-D4, LH24n-B95-D4, and LH20t-D4

		LH20t-D4	LH24n-D4	LH24n-B95-D4
all	MAE	1.76	1.87	1.74
	RMSD	2.45	2.42	2.38
	MSE	0.17	0.56	0.17
	MaxE(−)	-5.82	-8.19	-7.49
	MaxE(+)	6.77	5.08	5.99
	STDEV	2.47	2.38	2.39
only ΔE_{fwd}	MAE	1.43	1.52	1.38
	RMSD	1.97	1.89	1.81
	MSE	-0.19	0.06	-0.32
	MaxE(−)	-5.03	-3.80	-3.73
	MaxE(+)	5.15	3.77	4.44
	STDEV	2.00	1.92	1.82
only ΔE_{rev}	MAE	2.09	2.22	2.10
	RMSD	2.85	2.86	2.83
	MSE	0.54	1.07	0.65
	MaxE(−)	-5.82	-8.19	-7.49
	MaxE(+)	6.77	5.08	5.99
	STDEV	2.85	2.70	2.80
